# Dendritic crystallization in hydrous basaltic magmas controls magma mobility within the Earth’s crust

**DOI:** 10.1038/s41467-022-30890-8

**Published:** 2022-06-10

**Authors:** Fabio Arzilli, Margherita Polacci, Giuseppe La Spina, Nolwenn Le Gall, Edward W. Llewellin, Richard A. Brooker, Rafael Torres-Orozco, Danilo Di Genova, David A. Neave, Margaret E. Hartley, Heidy M. Mader, Daniele Giordano, Robert Atwood, Peter D. Lee, Florian Heidelbach, Mike R. Burton

**Affiliations:** 1 School of Science and Technology, Geology Division, University of Camerino, Camerino, Italy; 2 Department of Earth and Environmental Sciences, University of Manchester, Manchester, M13 9PL UK; 3 Istituto Nazionale di Geofisica e Vulcanologia-Osservatorio Etneo, Sezione di Catania, Catania, Italy; 4 Department of Mechanical Engineering, University College London, London, UK; 5 Research Complex at Harwell, Rutherford Appleton Laboratory, Harwell, Oxfordshire, 11 OX11 0FA UK; 6 Department of Earth Sciences, Durham University, Durham, DH1 3LE UK; 7 School of Earth Sciences, University of Bristol, Bristol, BS8 1RJ UK; 8 Centro de Ciencias de la Tierra, Universidad Veracruzana, Xalapa, 91090 Mexico; 9 Centre of Geosciences, National Autonomous University of Mexico, Queretaro, 76230 Mexico; 10 Bayerisches Geoinstitut, University of Bayreuth, 95440 Bayreuth, Germany; 11 Department of Earth Science, University of Torino, 10125 Torino, Italy; 12 Diamond Light Source, Harwell Science and Innovation Campus, Didcot, OX11 0DE UK

**Keywords:** Petrology, Volcanology

## Abstract

The majority of basaltic magmas stall in the Earth’s crust as a result of the rheological evolution caused by crystallization during transport. However, the relationships between crystallinity, rheology and eruptibility remain uncertain because it is difficult to observe dynamic magma crystallization in real time. Here, we present in-situ 4D data for crystal growth kinetics and the textural evolution of pyroxene during crystallization of trachybasaltic magmas in high-temperature experiments under water-saturated conditions at crustal pressures. We observe dendritic growth of pyroxene on initially euhedral cores, and a surprisingly rapid increase in crystal fraction and aspect ratio at undercooling ≥30 °C. Rapid dendritic crystallization favours a rheological transition from Newtonian to non-Newtonian behaviour within minutes. We use a numerical model to quantify the impact of rapid dendritic crystallization on basaltic dike propagation, and demonstrate its dramatic effect on magma mobility and eruptibility. Our results provide insights into the processes that control whether intrusions lead to eruption or not.

## Abstract (web summary)

In situ 4D experiments at high temperature and moderate pressure reveal that rapid dendritic crystallization in hydrous basaltic magmas promotes a rheological transition within minutes, controlling magma mobility within the Earth’s crust.

## Introduction

Crystallisation of basaltic magmas leads to rheological transitions, from Newtonian to non-Newtonian behaviour, which affect magma’s mobility within the crust^1–8^. Understanding crystallisation kinetics is therefore fundamental to understanding rheological transitions between eruptible, low-viscosity magmas and rigid mushes that stall at depth^9–14^.

Basaltic magmas generated in the Earth’s mantle can accumulate in the shallow crust (≤10 km depth), where they may experience periods of protracted stagnation in reservoirs before migrating within dikes and potentially erupting^12,15^. Magma mobility within the crust is affected by density contrasts and magma viscosity. Initially, when crystal content is low, magma viscosity is dominated by the contribution from the melt phase; as crystals grow, their contribution to rheology becomes increasingly important. Crystal growth kinetics are controlled by prevailing magmatic conditions (e.g., temperature, pressure, water content and oxygen fugacity), which in turn affect crystal fractions, sizes and shapes. Pyroxene, plagioclase and olivine are the major crystal phases in basaltic magmas. Their sizes and shapes record the physico-chemical processes that occur within the crust, during stagnation in reservoirs, and magma ascent within dikes^9,11,16–21^. In particular, the formation of crystal-rich magmas and/or crystal mushes at depth is part of the rheological evolution of magmas and may prevent them from reaching the Earth’s surface^9,12,13,17–19^. Previous studies have discussed the dynamics of crystal-rich magmas and crystal mushes under pre- and syn-eruptive conditions within dikes and reservoirs in active basaltic systems such as Mt Etna (Italy)^9–17^, Piton de la Fournaise (La Réunion)^13,19^, Haleakalā (Hawaii)^10,11,22^ and Bárðarbunga volcanic system (Iceland)^23^. These studies have highlighted how crystal mush formation promoted by fast dendritic crystal growth of pyroxene and olivine controls the eruptibility of some basaltic magmas^10,11,13,18^. Understanding the evolution of crystallisation kinetics and their effects on the rheology of basaltic magmas stored within the crust is fundamental for determining solidification timescales, and therefore constraining the mobility of magmatic bodies and the hazards associated with the potential eruptibility of basaltic systems.

Magma ascent towards the surface occurs mainly through dikes, which commonly develop as near-vertical features within the crust^15^. Dikes have the potential to either reach the surface and trigger an eruption, or to be frozen within the crust^24^. Whether or not a dike reaches the surface and produces an eruption depends on the flux of magma supplied from the magma reservoir, as well as the evolution of the physico-chemical properties of the magma and the mechanical properties of its host-rock. Crystallisation kinetics can have a major role on controlling the rheological behaviour of magma, promoting sudden and drastic transitions in magma rheology during dike propagation^9^.

Magma undercooling (*∆*T) controls both crystallisation kinetics and the evolution of crystal shape. ∆*T* is defined as the difference between the liquidus temperature, i.e. the highest temperature at which a specific mineral phase can crystallise, and the actual subliquidus temperature of magma at which crystallisation occurs^25,26^. Undercooling can result from either isobaric cooling at a fixed depth, or occur almost isothermally due to decompression on ascent and related loss of water (or a change in water activity) that results in an increase of the temperature of the liquidus. Crystal nucleation is delayed at low ∆*T* due to the high crystallisation energy barrier at conditions close to the liquidus. The style of growth is also affected by ∆*T* with a range from perfect euhedral crystal shapes to hopper/skeletal to swallowtail or dendritic shapes^27^. Dendritic crystal shapes are promoted by diffusion-limited growth at medium-to-high ∆*T*^11,16,28^. In basaltic volcanic systems, ∆*T* can range from few degrees up to hundreds of degrees Celsius^11,19,21,25,28–33^.

Clinopyroxene is a sensitive indicator of magma dynamics as its crystal shape is sensitive to changes in ∆*T*, and it can be used to determine the potential pre-eruptive conditions of basaltic volcanic systems^10,11,31–36^. The size, abundance and shape of clinopyroxene crystals in basaltic magmas can evolve in hours at a fixed set of conditions^30^ and in a few minutes during a rapid perturbation of undercooling^21^.

Dendritic crystallisation can occur rapidly, resulting in a rapid evolution of crystal shapes and the development of complex textures. These can be resolved directly in real time in three dimensions (3D) using in situ 4D (3D + time) X-ray computed microtomography (µCT) experiments. Until now these experiments have been restricted to anhydrous samples at atmospheric pressure, allowing investigation of crystallisation and its influence on magma rheology only at high temperatures and surface pressure^21,37,38^.

Here, we use a novel high-pressure experimental apparatus to perform in situ 4D µCT experiments at high temperatures and water-saturated conditions at crustal pressures, to investigate crystallisation kinetics and the 3D evolution of clinopyroxene shape in real time using a trachybasaltic melt from Mt Etna. In these experiments it is possible to quantify the crystal growth rate directly in 3D through time under water-saturated conditions at intermediate pressure. Consequently, we can investigate crystallisation kinetics and the morphological evolution of clinopyroxene as a function of ∆*T*, allowing us to quantify and model how crystallisation and the evolution of crystal aspect ratio can affect magma rheology under conditions relevant to crustal transport. We use a simple numerical dike propagation model^15,39^ to simulate magma ascent and test the effects of the observed systematics of dendritic crystallisation on magma rheology and mobility. This improved understanding of the potential eruptibility of a basaltic volcanic system will play an important role in hazard assessment and risk mitigation strategies in areas of active basaltic volcanism.

## Results and discussion

### 4D crystallisation experiments under water-saturated conditions

Experiments were performed in situ at beamline I12-JEEP, Diamond Light Source, Harwell, UK, using a hydrous trachybasaltic glass (Supplementary Table 1) from the 2001 Mt Etna eruption as the starting material (see Methods). To simulate magmatic pressures and temperatures within the shallow crust, the P2R uniaxial mechanical rig^40,41^ was combined with a high-temperature environmental cell (Alice furnace^42^) (Supplementary Fig. 1). These two apparatuses combined with fast synchrotron X-ray microtomography allowed us to capture the evolution of crystallisation in a trachybasaltic melt with acquisition of a 3D volume every 60 s at ~3 µm resolution.

**Fig. 1:**
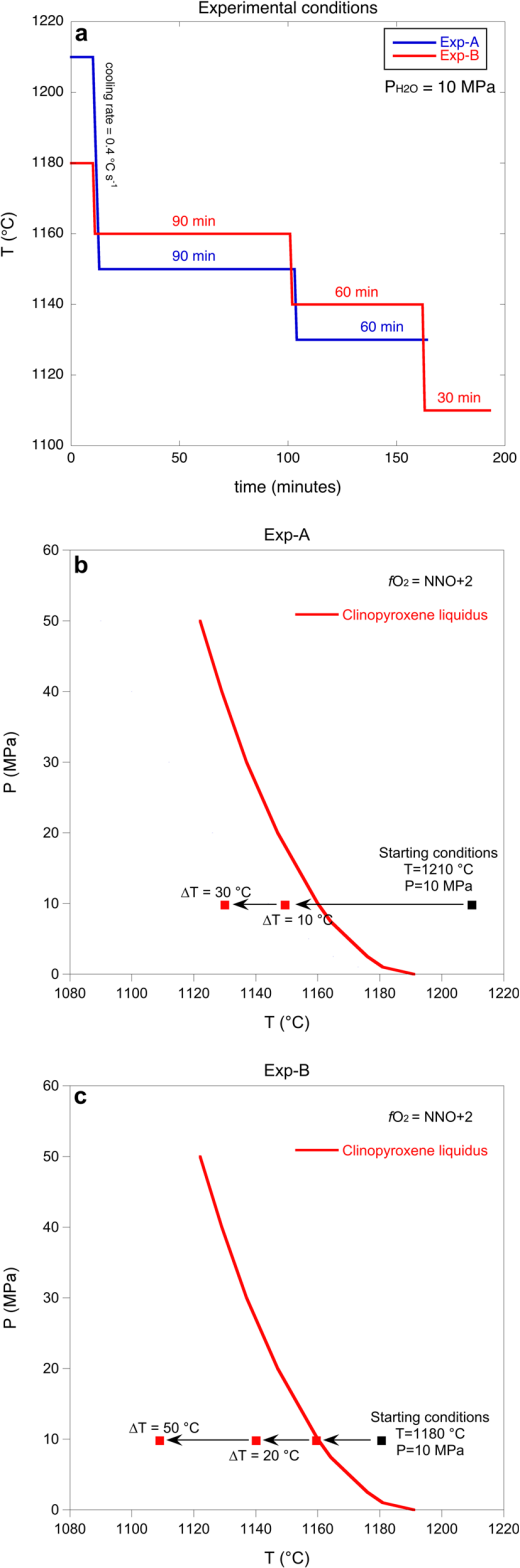
Experimental conditions. **a** The hydrous samples were heated to 1210 °C (Exp-A) and 1180 °C (Exp-B) and they were held for 10 min at these temperatures above the liquidus (**b**, **c**). Pressure was maintained constant at 10 MPa (**a**–**c**). **a**, **b** The initial temperature of Exp-A (1210 °C) was decreased to 1150 °C for 90 min and then to 1130 °C for 60 min. **a**, **c** The initial temperature of Exp-B (1180 °C) was decreased to 1160 °C for 90 min, then to 1140 °C for 60 min and, finally, to 1110 °C for 30 min. **b**, **c** Clinopyroxene liquidus is obtained with Rhyolite-MELTS software (version 1.2), using the 2001 Etna trachybasaltic composition (Supplementary Table 1) under water-saturated conditions. The undercoolings (∆*T*) investigated are calculated with respect to the clinopyroxene liquidus.

Two single-step cooling experiments (Exp-A and Exp-B) were performed at constant pressure (~10 MPa), degree of water-saturation (~0.94 wt.%) and at an oxygen fugacity of NNO + 2 (Fig. 1) in order to simulate the stagnation of a hydrous basaltic magma in a reservoir within the crust. Experiments were initially held above the ~1160 °C liquidus for 10 min, specifically at 1210 °C (Exp-A) and 1180 °C (Exp-B) (Fig. 1). Temperature was then rapidly reduced at a rate of ~0.4 °C s^−1^ to different sub-liquidus temperatures and held constant for given amounts of time to investigate the role of ∆*T* on crystallisation kinetics and the morphological evolution of clinopyroxene crystals. The temperature of Exp-A was decreased from 1210 °C to 1150 °C (i.e. at a ∆*T* of 10 °C) and kept at that temperature for 90 min before being reduced to 1130 °C (i.e. at a ∆*T* of 30 °C) and being held for further 60 min (Fig. 1 and Supplementary Table 2). The temperature of Exp-B was instead decreased from 1180 °C to 1160 °C and kept at a constant temperature for 90 min before being decreased to 1140 °C (i.e. at a ∆*T* of 20 °C) and held for further 60 min, and then finally decreased to 1110 °C (i.e. at ∆*T* of 50 °C) and maintained at this temperature for another 30 min (Fig. 1 and Supplementary Table 2).

**Fig. 2:**
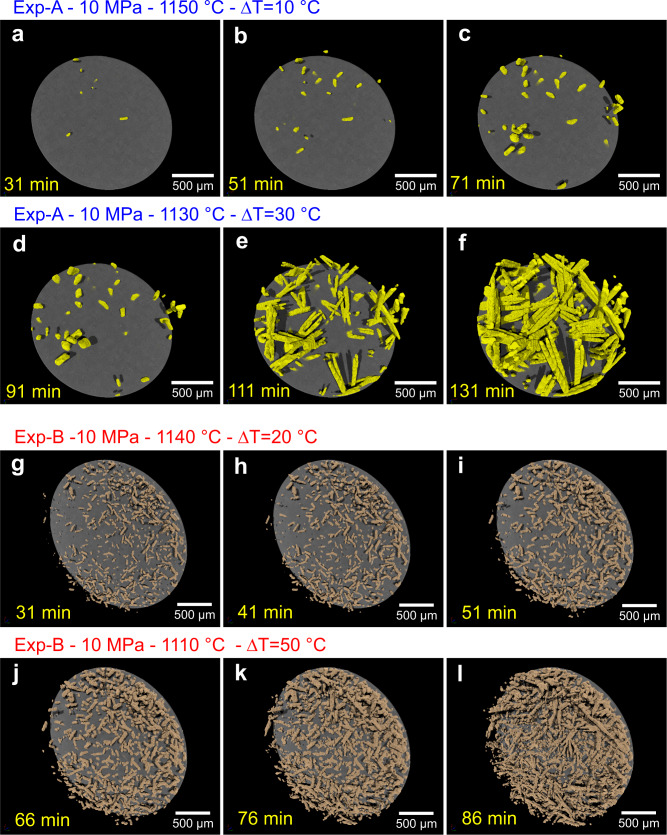
Crystallisation of a hydrous trachybasaltic melt through time during single-step cooling experiments at 10 MPa. **a**–**c** Volume renderings show clinopyroxene crystallisation through time during the experiment Exp-A at 1150 °C and ∆*T* = 10 °C. **d**–**f** Volume renderings show clinopyroxene crystallisation through time during the experiment Exp-A at 1130 °C and ∆*T* = 30 °C. **g**–**i** Volume renderings show clinopyroxene crystallisation through time during the experiment Exp-B at 1140 °C and ∆*T* = 20 °C. **j**–**l** Volume renderings show clinopyroxene crystallisation through time during the experiment Exp-B at 1110 °C and ∆*T* = 50 °C. **a**–**l** The undercoolings (∆*T*) investigated are calculated with respect to the clinopyroxene liquidus. The time in yellow reported in each panel indicates the time passed from the beginning of the experiment.

Clinopyroxene (En_39–41_, Fs_10–11_, Wo_48–50_; Supplementary Fig. 2) is the first crystallising phase in both experiments, in terms of both time and undercooling. During the first 90 min, clinopyroxene nucleated at 1150 °C in Exp-A, but it did not nucleate at 1160 °C in Exp-B (Fig. 2 and Supplementary Fig. 3). This liquidus temperature of ~1160 °C and clinopyroxene crystallisation is consistent with Rhyolite-MELTS^43^ under water-saturated conditions at 10 MPa and an oxygen fugacity of NNO + 2 (Fig. 1).

### Interface-controlled growth and euhedral shapes

**Fig. 3:**
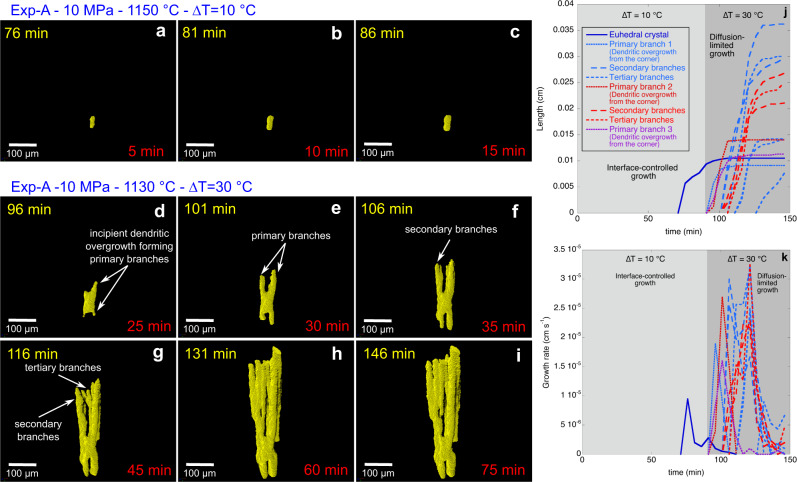
Crystallisation and crystal shape evolution of a clinopyroxene crystal through time during the experiment Exp-A. **a**–**c** Volume renderings show the growth of a euhedral clinopyroxene crystal through time at 1150 °C and ∆*T* = 10 °C. **d**–**i** Volume renderings show the transition between euhedral to dendritic shapes and the growth of a dendritic clinopyroxene crystal through time at 1130 °C and ∆*T* = 30 °C. The formation of primary branches indicates the transition from an interface-controlled growth to a diffusion-limited growth (**d**, **e**). Secondary (**f**) and tertiary (**g**) branches are formed through time under diffusion-limited growth. **j** Evolution of the maximum length (along c axis) of a clinopyroxene crystal through time at ∆*T* = 10 °C and ∆*T* = 30 °C. **k** Growth rate of a clinopyroxene crystal as a function of time at ∆*T* = 10 °C and ∆*T* = 30 °C. **a**–**k** The undercoolings (∆*T*) investigated are calculated with respect to the clinopyroxene liquidus. **a**–**i** The time in yellow reported in each panel indicates the time passed from the beginning of the experiment. The time in red reported in each panel indicates the time passed from the formation of the pyroxene crystal.

**Fig. 4:**
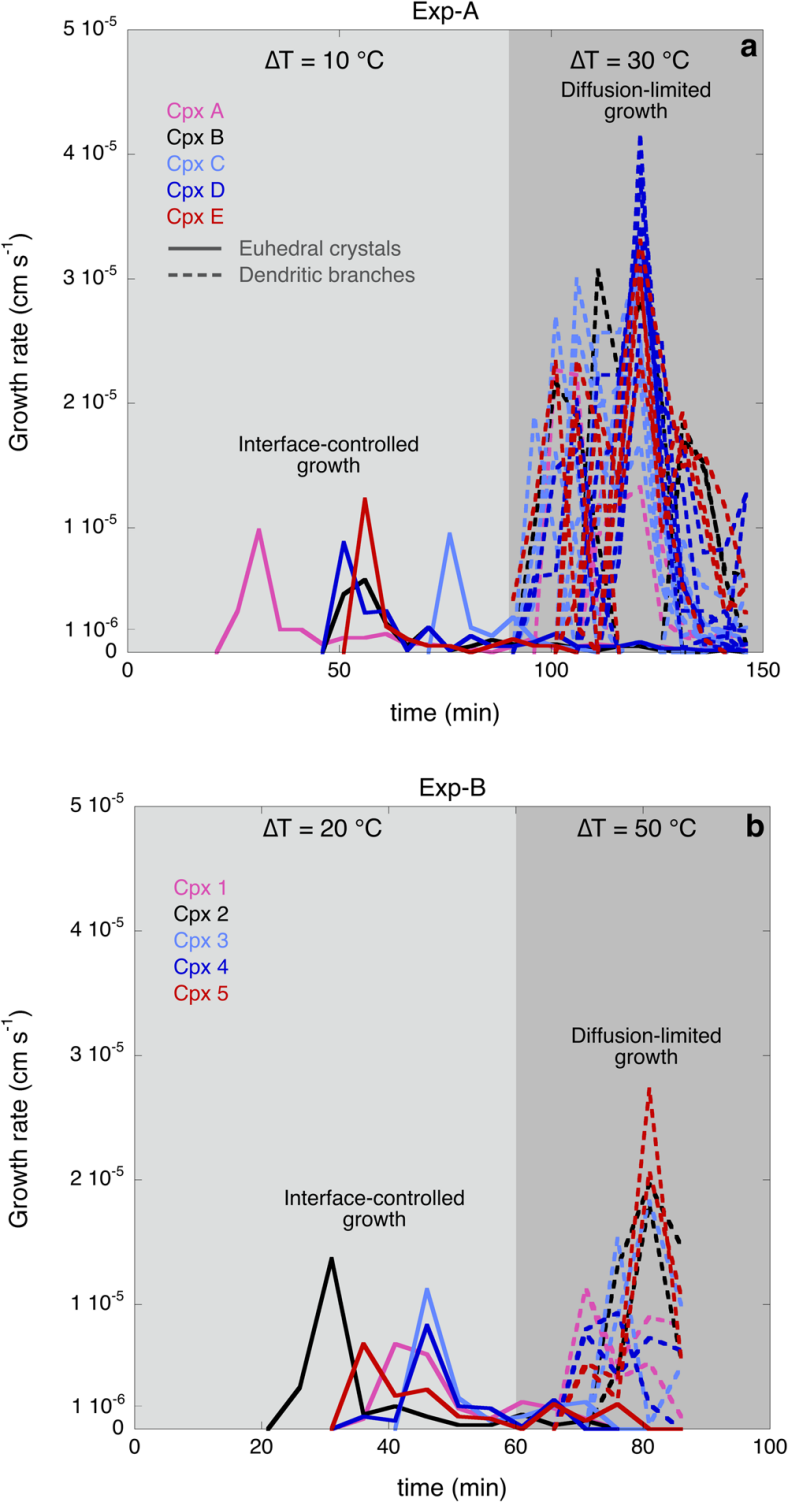
Growth rate of clinopyroxene crystals as a function of the experimental time. **a** Plot showing the evolution of growth rates of 5 clinopyroxene crystals through time during the experiment Exp-A at ∆*T* = 10 °C (euhedral crystal shapes are promoted by interface-controlled growth) and at ∆*T* = 30 °C (dendritic crystal shapes are promoted by diffusion-limited growth). Light grey shading represents ∆*T* = 10 °C, dark grey shading represents ∆*T* = 30 °C. **b** Plot showing the evolution of growth rates of 5 clinopyroxene crystals through time during the experiment Exp-B at ∆*T* = 20 °C (euhedral crystal shapes are promoted by interface-controlled growth) and at ∆*T* = 50 °C (dendritic crystal shapes are promoted by diffusion-limited growth). Light grey shading represents ∆*T* = 20 °C, dark grey shading represents ∆*T* = 50 °C. The undercoolings (∆*T*) investigated are calculated with respect to the clinopyroxene liquidus.

Once temperature was decreased to subliquidus conditions, the onset of clinopyroxene crystallisation occurred after 21 min at 1150 °C (∆*T* = 10 °C) during experiment Exp-A and after 5 min at 1140 °C (∆*T* = 20 °C) during experiment Exp-B. The small ∆*T* investigated (10 and 20 °C) during the first parts of experiments Exp-A and Exp-B generated distinctive blocky and euhedral clinopyroxene crystals (Figs. 2a–c, g–i and 3a–c; and Supplementary Fig. 3), suggesting growth was interface-controlled. Interface-controlled growth promotes homogeneous molecular attachment along crystal surfaces of pre-formed crystals, as the uptake rate of components at the crystal-melt interface (crystal growth rate) is slower than the diffusion rate of chemical components within the melt, leading to the formation of euhedral shapes^25,26^. Our 4D experimental results indicate that interface-controlled growth and the formation of euhedral clinopyroxene crystals occurs within a narrow range of undercooling (∆*T* $\le$ 20 °C), which is in agreement with previous studies^11,44^. During the 90 min at 1150 °C (∆*T* = 10 °C; Exp-A) and the 60 min at 1140 °C (∆*T* = 20 °C; Exp-B), several nucleation events were observed through time (Figs. 2 and 4). This implies that the nucleation of new crystals under constant temperature and pressure conditions is not restricted to a single event. This was also noted in experiments on anhydrous basaltic melts^37^ and hydrous trachytic melts^45^. Our 4D observations therefore show that both crystal nucleation and growth occurred throughout our experiments (Fig. 4).

**Fig. 5:**
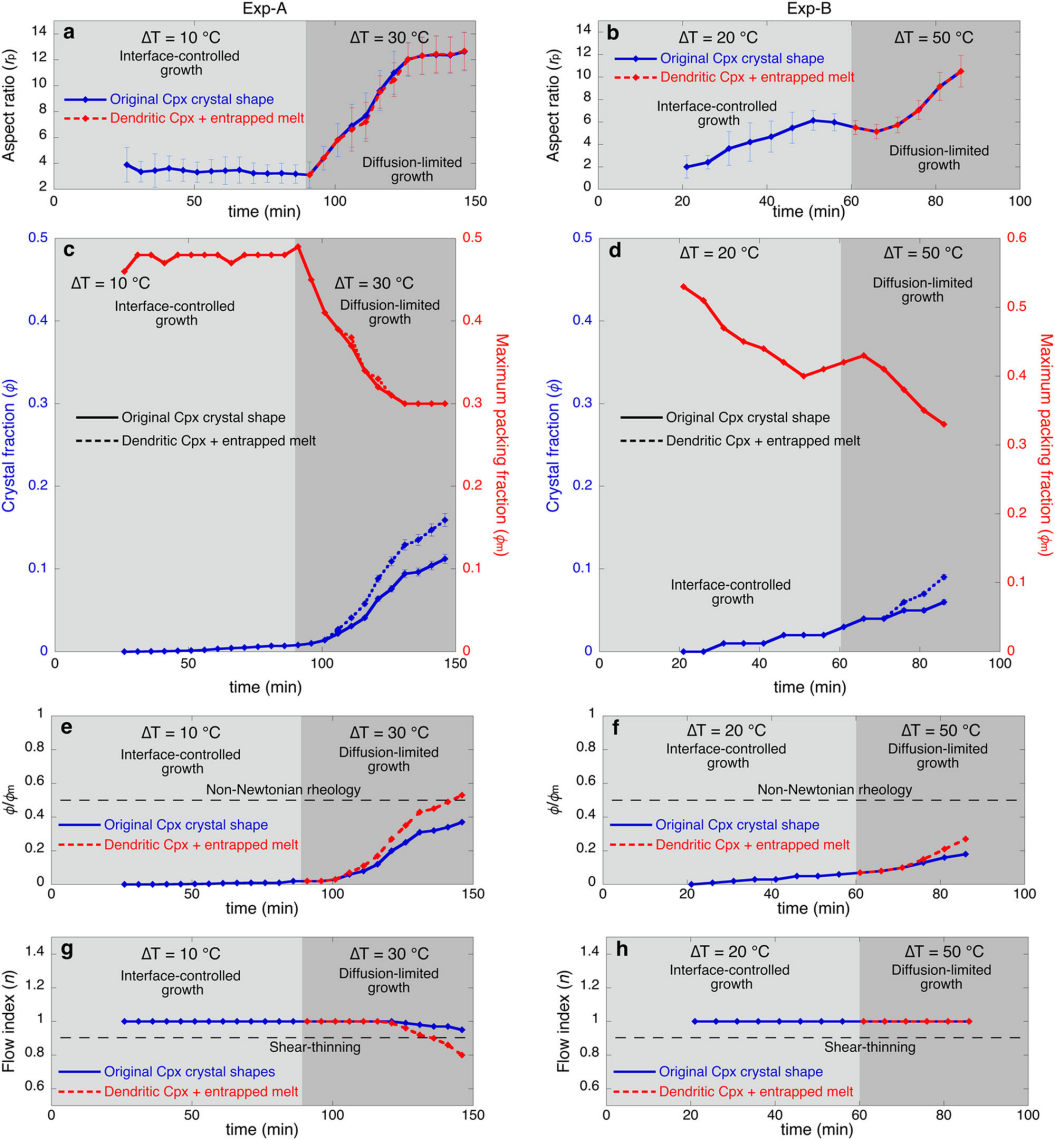
Rheological transition of a trachybasaltic magma during crystallisation. **a**, **b** Evolution of clinopyroxene aspect ratio (*r*_p_) through time as a function of ∆*T* (undercooling) during the experiments Exp-A (**a**) and Exp-B (**b**). **c**, **d** Evolution of clinopyroxene crystal fraction (*ϕ*) through time as a function of ∆*T* during the experiments Exp-A (**c**) and Exp-B (**d**). Diffusion-limited growth, promoted at ∆*T* = 30 °C (Exp-A) and ∆*T* = 50 °C (Exp-B), favours dendritic crystal shapes which entrap melt within crystal branches. The entrapped melt within the original dendritic crystals (blue) is considered an additional solid part, which contributes to further increase the crystal volume of the dendritic crystals (red), increasing consequently the crystal volume fraction (**a**, **b**). **c**–**h** Evolution of the maximum packing fraction (*ϕ*_m_), ratio *ϕ*/*ϕ*_m_ and flow index ($n$) which are indicators of the rheological behaviour of a crystal-bearing magma (Mader et al.). **e**, **f** Newtonian rheology is promoted at relatively low *ϕ/ϕ*_m_ (<0.5), whereas non-Newtonian rheology, characterised for example by shear thinning, is more pronounced as *ϕ*/*ϕ*_m_ increases. Shear thinning behaviour is promoted when $n$ <1. **a**, **c**, **e**, **g** Light grey shading represents ∆*T* = 10 °C, dark grey shading represents ∆*T* = 30 °C. **b**, **d**, **f**, **h** Light grey shading represents ∆*T* = 20 °C, dark grey shading represents ∆*T* = 50 °C.

In Fig. 4 we report five representative crystals, for which we can track the evolution of euhedral and dendritic shapes, the latter with several generations of branches. Both experiments have initial individual blocky euhedral clinopyroxene crystals showing similar growth rate (*Y*_L_) profiles, in which *Y*_L_ ranges between 10^−7^ and 10^−5 ^cm s^−1^ (Fig. 4). These unique observations of the continuous growth of clinopyroxene crystals at magmatic conditions show that the growth rate profile of a single crystal initially accelerates rapidly from 10^−7 ^cm s^−1^ to a maximum of 10^−5 ^cm s^−1^. After passing the peak of maximum growth, growth rate decreases to 10^−7 ^cm s^−1^ as the crystal approaches its final, equilibrium size (Fig. 4). The shape of the growth rate profile for each crystal is similar through time, which indicates that the crystals that nucleate later in time can grow with similar growth rates (10^−7^−10^−5^ cm s^−1^) to the crystals nucleated earlier, and that they can also reach similar sizes. The rheological evolution of a trachybasaltic magma during crystallisation of euhedral crystals is reported in Fig. 5a–h. The evolution of euhedral shapes from nucleation to near-equilibrium crystal sizes (with maximum length of ~100 µm) takes between 40 and 70 min. Although rapid growth is recorded by euhedral clinopyroxenes along their long axes (Figs. 3 and 4), the range of aspect ratios (*r*_p_ from 3.22 to 3.89) and crystal fractions (*ϕ* up to 0.007) at ∆*T* = 10 °C after 90 min are relatively small (Fig. 5a, c). A larger range of aspect ratios (*r*_p_ 2.05–6.14) is observed at ∆*T* = 20 °C (Fig. 5b) where *ϕ* reaches 0.02 after 60 min (Fig. 5d).

### Diffusion-limited growth and dendritic shapes

At larger undercoolings (Exp-A: 30 °C of ∆*T*; Exp-B: 50 °C of ∆*T*), we observe diffusion-limited crystal growth and the formation of dendritic shapes (Figs. 2 and 3). Crystal growth is controlled by diffusion when the attachment kinetics are faster than diffusion in the melt^25,26,29,46^. This agrees with previous experimental results which document the formation of dendritic clinopyroxene at ∆*T* ≥ 30 °C in anhydrous^37,44^ and hydrous basaltic magmas^31,32^. In general, euhedral crystal shapes cannot be generated under high ∆*T*s^29^. Therefore, when ∆*T* increases, primary branches begin to develop at the corners of euhedral crystals promoting crystal overgrowth^11,46,47^ (Fig. 3d, e and Supplementary Movie 1). Branching typically starts at the corners of crystals during the onset of the transition from euhedral to dendritic shapes. During the first stage of the overgrowth, incompatible elements can accumulate around the primary branches (Fig. 3d, e), leading to the continued overgrowth of secondary and tertiary branches^46^ (Fig. 3f–k). Electron backscatter diffraction (EBSD) data indicate that the vast majority of the clinopyroxene crystals have a low internal misorientation (between 0 and 2°); only some crystals show a continuous lattice bending up to 3°. Rarely, larger stepwise misorientations between host crystal and branch in the form of a subgrain boundary are observed (Supplementary Fig. 4). This indicates that crystallographic branching was the preferred growth mechanism during overgrowth of existing euhedral crystals with minor non-crystallographic branching. The dendritic morphology observed in our experiments is therefore the result of continued overgrowth of existing grains, by crystallographic and non-crystallographic branching during diffusion-limited growth.

The branching triggers rapid dendritic growth of the crystals at rates of ~10^−5 ^cm s^−1^ (Fig. 3j, k), resulting in crystals with elongated dendritic shape. The growth rate profile of dendritic branches is similar to the euhedral crystals, in which we observed a rapid increase of the growth rate up to a peak and then a subsequent decrease. However, individual dendritic branches grow a factor of 2–4 faster than euhedral crystals, reaching up to 4 × 10^−5 ^cm s^−1^ (Fig. 4), which promotes a rapid change in crystal sizes (e.g., maximum length) and volume fractions.

Clinopyroxene crystals grown at ∆*T* = 30 °C reached ~400 µm in length in 60 min. In previous studies, large crystals have traditionally been considered to grow in a tree-ring fashion, in which the oldest part of the crystal lies at the centre and the youngest at the rim^48,49^. However, our results illustrate that rapid growth promoted at ∆*T* = 30 °C generates elongated dendritic crystal frameworks that can be progressively infilled by further crystal growth, as the growth rate decreases. The crystal growth fashion observed in the in situ 4D experiments therefore has important implications for the entrapment of melt inclusions and for constraining the growth chronology of crystals^16,19,20,49,50^.

### Rheological transition during dendritic crystallisation

The quantification of crystallisation kinetics presented above allows us to infer the impact of crystal growth on magma rheology and magma mobility within the crust. Previous work has shown that crystal shape – particularly aspect ratio – and crystal fraction are the first order textural controls on suspension rheology^1,4–8,51–53^. Magma rheology is also modulated by other variables such as the dispersion of crystal sizes^53^, presence of bubbles^6,54^, cooling rate^55^ and oxygen fugacity^56^. Here we model the effect of crystal shape and aspect ratio on magma rheology using the parameterisation proposed by Mader et al.^6^, based on the formulation of Krieger and Dougherty^57^:

1 $${\eta }_{{{{{{\rm{r}}}}}}}=1-{\left(\tfrac{\phi }{{\phi }_{{{{{{\rm{m}}}}}}}}\right)}^{{{{{{\rm{B}}}}}}{\phi }_{{{{{{\rm{m}}}}}}}},$$

where *η*_r_ is the relative apparent viscosity, *ϕ* is the crystal fraction, *ϕ*_m_ is the maximum packing fraction and B is the Einstein coefficient.

The maximum packing fraction (*ϕ*_m_) represents the critical volume fraction beyond which the suspension becomes rheologically locked, and it is calculated for both experiments following Mader et al. ^6^:

2 $${\phi }_{{{{{{\rm{m}}}}}}}=0.55\,{{\exp }}[-\tfrac{{({{{\log }}}_{10}{r}_{{{{{{\rm{p}}}}}}})}^{2}}{2}],$$

where *r*_p_ is particle aspect ratio (long over short axis). Newtonian rheology is promoted at relatively low *ϕ*/*ϕ*_m_ (<0.5), whereas non-Newtonian rheology, characterised for example by shear thinning, occurs when *ϕ*/*ϕ*_m_ increases^6^. For a shear thinning suspension, the relative apparent viscosity becomes dependent on the strain rate $\dot{{{{{{\rm{\gamma }}}}}}}$, yielding (following Mader et al.^6^):

3 $${\eta }_{{{{{{\rm{r}}}}}}}=\left[1-{\left(\tfrac{\phi }{{\phi }_{{{{{{\rm{m}}}}}}}}\right)}^{{{{{{\rm{B}}}}}}{\phi }_{{{{{{\rm{m}}}}}}}}\right]{\dot{{{{{{\rm{\gamma }}}}}}}}^{{{{{{\rm{n}}}}}}-1},$$

where $n$ is the flow index, which depends on both aspect ratio and on *ϕ*/*ϕ*_m_, according to the following empirical relationship^58^:

4 $$n=1-0.2{r}_{{{{{{\rm{p}}}}}}}\,{\left(\tfrac{\phi }{{\phi }_{m}}\right)}^{4}.$$

Shear thinning behaviour occurs when *n* < 1, and when *n* < 0.9, shear thinning cannot be neglected^58^.

Our 4D textural characterisation allows us to evaluate crystal fraction (*ϕ*) and aspect ratio (*r*_p_) through time, and therefore to calculate the maximum packing fraction (*ϕ*_m_), the ratio *ϕ*/*ϕ*_m_ and the flow index ($n$) in order to evaluate the rheological behaviour of the crystal-bearing magma. Our results show that aspect ratio is almost constant at ~3 (Fig. 5a) during 90 min at ∆*T* = 10 °C (Fig. 5e and Supplementary Table 3), and increases from 2.05 to 6.14 (Fig. 5b) during 60 min at ∆*T* = 20 °C (Fig. 5f and Supplementary Table 3). This corresponds to an almost constant *ϕ*_m_ ≈ 0.40 in the first case, and a decrease from *ϕ*_m_ = 0.53 to *ϕ*_m_ = 0.40 in the second case. In both cases *n* is very close to 1 throughout, hence we infer that a static hydrous basaltic magma at ∆*T* between 10 and 20 °C exhibits Newtonian rheology and no rheological transition is promoted by progressive interface-controlled growth of clinopyroxene (Fig. 5g, h).

Dendritic growth promotes a rapid increase in the maximum length of the pyroxene crystals (Fig. 4), as the growth of the dendritic branches is preferentially oriented along the long crystallographic axis. This strongly increases the average aspect ratio from 3.11 to 12.67 in <60 min at ∆*T* = 30 °C (Fig. 5a and Supplementary Table 3) and, from 5.50 to 10.52 in 30 min at ∆*T* = 50 °C (Fig. 5b and Supplementary Table 3). Dendritic growth also favours the entrapment of melt between branches. The entrapped melt is stagnant and therefore effectively increases the non-deformable portion of the magmatic mixture. Thus, we considered the entrapped melt as an active rigid component that enhances the crystal fraction of magma. As a consequence, the effect of dendritic crystal growth has an enhanced impact on magma rheology via two mechanisms: (1) by increasing the effective crystal fraction above that of the crystal phase alone, through trapping of melt; and (2) by decreasing the maximum packing fraction through formation of high aspect ratio crystals. Progressive dendritic growth at high undercooling therefore progressively decreases the flow index values (Fig. 5c–h and Supplementary Table 3), and we find that the effect of entrapped melt on the effective rheological properties of moving magmas and on rheological transitions can be significant (Fig. 5). Henceforth, rheological calculations and discussions related to dendritic crystallisation are based on the crystal fraction calculated considering the melt entrapped within dendritic crystals.

Diffusion-limited dendritic crystal growth in Exp-A triggered a rapid increase of *ϕ* from 0.008 to 0.16 in 60 min at ∆*T* = 30 °C (Fig. 5c). Because of the rapid increase of crystal aspect ratios (Fig. 5a), the maximum packing fraction (*ϕ*_m_) decreased from 0.49 to 0.30 (Fig. 5c). Therefore, a simultaneous increase of *ϕ* and decrease of *ϕ*_m_ resulted in a rapid approach towards the maximum packing fraction (Fig. 5c). Large aspect ratios (*r*_p_ ~12) and the increase of *ϕ* produced by dendritic crystallisation during the last 20 min of Exp-A increased *ϕ*/*ϕ*_m_ above 0.5 (Fig. 5e) and decreased the flow index below 0.9 (Fig. 5g). These results indicated that a rheological shift from Newtonian to non-Newtonian behaviour was induced within 60 min at ∆*T* = 30 °C.

A larger undercooling was investigated in Exp-B (∆*T* = 50 °C) than in Exp-A in order to study the effect of undercooling degree on crystallisation kinetics and if the transition from euhedral to dendritic shapes affects rheology over shorter timescales (30 min instead of 60 min). During 30 min at ∆*T* = 50 °C, a rapid increase of the aspect ratio was observed (~5–~11) (Fig. 5b) alongside a decrease in *ϕ*_m_ from 0.43 to 0.33 (Fig. 5d). The crystal fraction increased from 0.03 to 0.09, particularly during the last 20 min of the experiment when dendritic crystallisation was dominant (Fig. 5d), resulting in a corresponding increase in *ϕ*/*ϕ*_m_ (Fig. 5f). However, after 30 min, *ϕ*/*ϕ*_m_ was smaller than 0.5 and $n$ was very close to 1 (Fig. 5f, h), which is indicative of Newtonian behaviour. Therefore, dendritic clinopyroxene crystallisation at ∆*T* = 50 °C is not able to promote a rheological transition to non-Newtonian behaviour within 30 min (Fig. 5d, f, h) at pressure of 10 MPa under water-saturated conditions. This implies that although a larger undercooling (∆*T* = 50 °C) was investigated in Exp-B compared to Exp-A (∆*T*$\le$30 °C), dendritic crystallisation kinetics are similar and there is no acceleration towards a rheological transition upon shorter crystallisation timescales. At ∆*T* = 50 °C, dendritic crystallisation may lead to a rheological transition after at least 30 min, as the equilibrium clinopyroxene crystal fraction is expected to be ~0.21 (according to Rhyolite MELTS and Moschini et al.^32^), which may favour a further increase of *ϕ*/*ϕ*_m_ and a decrease of the flow index.

The increase of ∆*T* from 10 °C to 30 °C in Exp-A affected the aspect ratio within the first ~5 min, whilst increasing ∆*T* from 20 °C to 50 °C in Exp-B did not dramatically affect either the aspect ratio or the crystal fraction within the first ~10 min. After 10 min, however, we observed an increase in both aspect ratio and crystal volume fraction. The delay in the aspect ratio response to the variations in undercooling was caused by the re-organisation of crystal shape driven by the shift from interface-controlled growth to diffusion-limited growth. Therefore, dendritic crystallisation was initially delayed in both experiments, but to different extents, as an increase of 30 °C of ∆*T* (from 20 to 50 °C in Exp-B) required a slightly longer delay for the transition to diffusion-limited growth than an increase of 20 °C (from 10 to 30 °C in Exp-A). After the first 10 min at higher ∆*T*s (30 and 50 °C in Exp-A and Exp-B, respectively), dendritic crystallisation became dominant, producing rapid increases in aspect ratio and crystal fraction (Fig. 5a–d). The transition delay from interface-controlled growth to dendritic growth is a clear example of how disequilibrium affects crystallisation and rheology in basaltic magmas, and consequently, magma dynamics at depth. These 4D experiments provide a unique insight into the disequilibrium effects on the evolution of aspect ratio and crystal fraction during dendritic growth under realistic magmatic conditions (Fig. 5a–d), elucidating how dendritic crystallisation can promote a rheological transition from Newtonian to non-Newtonian rheology as a function of undercooling and time (Fig. 5e–h). Results from previous ex-situ rheological experiments on basaltic and andesitic magmas corroborate our findings on the role of dendritic crystallisation on rheological transitions in magmas, showing that relatively high aspect ratios (7–13) of dendritic plagioclase crystals yield a shear-thinning behaviour at crystal fractions as low as ~0.13^4,7,51,52^.

### Dendritic crystallisation and dike propagation: implications for magma mobility and eruptibility

Recent studies on magmas from Mt Etna have demonstrated that dikes can either form intrusive bodies at 100-300 m depth^9^ or feed lava-flow and lava-fountaining eruptions like the dike-fed July 2001 eruption^59,60^. It has been shown that magma transport through dikes, and magma convection within sills, is affected by variations in thermal gradients, cooling rates and crystallisation regimes (such as interface-controlled versus diffusion-limited crystal growth), and that such variations can reduce the mobility of basaltic magmas in the shallow crust^9,17,18,61^.

Our in situ 4D experiments indicate that dendritic crystallisation of clinopyroxene can be triggered by moderate ∆*T*s (≥ 30 °C) within relatively short timescales (minutes). Previous work has also demonstrated that diffusion-limited crystallisation of plagioclase in basaltic melts promotes elongated dendritic and skeletal crystal habits at ∆*T*s ≥ 25 °C^4,21,38^, with aspect ratios between 7 and 9^4,38,52^, comparable to those of our 4D experiments, and reaching higher aspect ratios (~13) at high ∆*T*s^21,51^. This implies that, at values of ∆*T* > 30 °C, a rapid transition in crystal shape can occur, potentially resulting in a transition from Newtonian to non-Newtonian rheological behaviour. This rheological transition and the rapid approach towards the maximum packing fraction will inhibit magma mobility within the crust. To investigate the role of crystallisation on the rheology and, thus, mobility of basaltic magmas, we simulated the propagation of a vertically oriented dike connected to a magma reservoir^15,39^ coupled with appropriate crystallisation^62–64^ and rheological^6^ models. Specifically, the crystallisation model used in this study was developed by La Spina et al.^63^ for the Etna 2001 eruption (lower vents). The model^63^ calculates the crystal content at each pressure, temperature and water content using a polynomial fitting derived by Rhyolite-MELTS^43^. Because we are interested in the effect of crystallisation kinetics on magma rheology within a basaltic dike, we assumed that viscous dissipation is dominant, and thus the resistance of the host rock to fracture can be neglected^15,39^. Therefore, the driving force for dike propagation is magma buoyancy, whilst the dominant resistance to propagation is the viscous flow of the magma^39^. With these assumptions, the half-width of the dike tail h^*^ can be calculated as follows:

5 $${h}^{* }={\left(\frac{3\mu {{{{{\rm{Q}}}}}}}{2\triangle \rho {{{{{\rm{g}}}}}}}\right)}^{1/3}$$

where *µ* is magma viscosity, Q is the 2D volumetric flux of magma injected into the propagating dike and ∆*ρ* is the difference between the crustal host-rock density and the magma density^39^. It is important to highlight that by using this model we do not want to simulate or describe how dike propagates within the crust, but we want to highlight how dendritic crystallisation affects magma rheology during dike ascent.

We simulated dike propagation using the trachybasaltic composition of the dike intrusion that resulted in the Mt Etna 2001 eruption^59^, the same as our 4D experiments starting material. We assumed that a trachybasaltic magma under water-saturated conditions is injected at 10 km depth into the host-rock at 1130 °C (close to the liquidus temperature of the whole rock). Estimates of dike propagation speeds, deduced from seismic observations, are in the order of metres per second^24^. We investigated an initial propagation speed of ~1 ms^−1^ corresponding to a dike of 20 cm width at 10 km depth^24,65^. During dike propagation towards the surface, ascending magma cools down, due to the thermal gradient at the wall of the dike^9,66^, and adiabatic cooling^62^. At the same time, magma temperature increases due to the release of latent heat of crystallization^62,67^. Since in our dike propagation model the conservation equation of energy is not solved, the net effect of adiabatic cooling and latent heat during crystallization is considered assuming different constant cooling rates, from 10^−4^ to 10^−3^ °C s^−1^, which are similar to those of effusive basaltic eruptions (in which magma ascent is a few metres per second) obtained from La Spina et al.^64^. The lateral cooling resulting from heat dissipation with the wall of the dike during static conditions^9^ is assumed to be negligible during magma ascent^9,64^ and ignored in the simulations. However, such cooling could have a role when dike propagation slows significantly, as ∆*T* can further increase from the innermost to the outermost part of the dike further promoting dendritic crystallization^9^. We also simulated a scenario in which adiabatic cooling was neglected, and the temperature was therefore constant along the dike (i.e., cooling rate = 0).

**Fig. 6:**
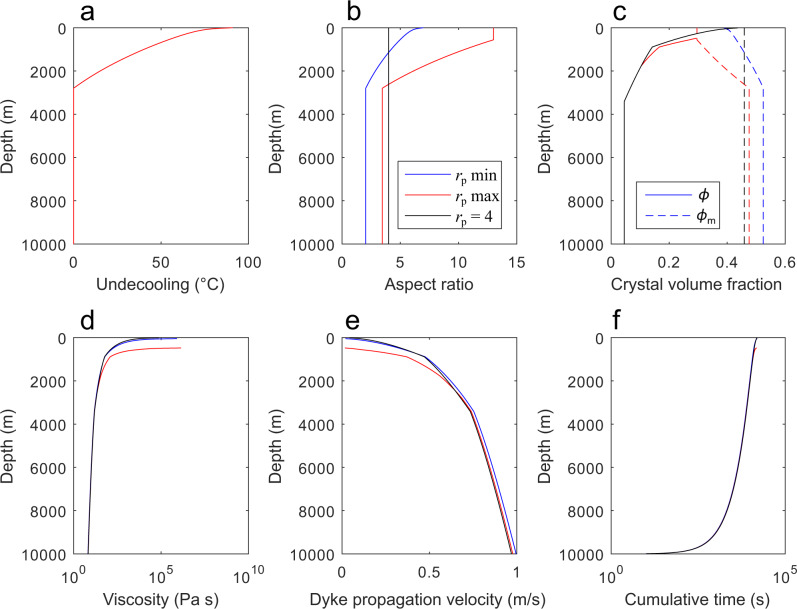
Model results during dike propagation towards the surface. Plots of magma ascent dynamics within a dike in which the cooling rate during ascent is 2 × 10^−3^ °C s^−1^. **a** Undercooling (∆*T*) as a function of depth. **b** Crystal aspect ratio as a function of ∆*T*. **c** Crystal volume fraction as a function of ∆*T*. The solid lines represent the crystal volume fraction (*ϕ*), whereas the dashed lines represent the maximum packing fraction (*ϕ*_m_). The red line represents the maximum aspect ratios achievable at ∆*T* ≥ 30 °C during dendritic crystal growth and considers the entrapment of melt between branches that further increases the crystal volume fraction. **d** Magma viscosity as a function of depth. **e** Dike propagation velocity as a function of depth. **f** Time needed to propagate dike from 10 km to the depths where the *ϕ*_m_ is met. Data plotted in Fig. 6 are provided in the Supplementary Data 1.

For our simulations we considered three different crystal growth scenarios in order to quantify the effect of the crystal aspect ratio on magma rheology during ascent (Fig. 6a–f): (i) in the first case we assumed an interface-controlled crystal growth at a constant aspect ratio (*r*_p_ = 4, black lines in Fig. 6a–f), (ii) in the second case we considered the transition from interface-controlled to diffusion-limited growth at ∆*T* ≥ 30 °C (blue line) and (iii) finally, we assumed that aspect ratio evolved as a function of ∆*T* (red line). We modelled two different profiles of aspect ratio (blue and red lines in Fig. 6b): the first considered the minimum aspect ratios achievable at ∆*T* ≥ 30 °C, simulating disequilibrium during the evolution of the dendritic crystal shape (blue line in Fig. 6b); the second assumed that dendritic growth had enough time to reach the maximum aspect ratio (red line in Fig. 6b), and it also considered the entrapment of melt between branches that further increased the crystal volume fraction (as shown in Fig. 5). By modelling these two types of aspect ratio evolution we covered the two end-member scenarios of dike ascent, one at strong disequilibrium (or rapid dike propagation), and the other close to equilibrium (or slow dike propagation).

In Fig. 6 we illustrate the magma ascent dynamics within a dike in which, for example, the cooling rate during ascent is 2 × 10^-3^ °C s^−1^. Crystallization mostly occurred during magma ascent at depths shallower than 3 km (Fig. 6c). If we consider the transition from interface-controlled to diffusion-limited growth (Fig. 6, blue and red line) as a function of ∆*T* (where dendritic growth is promoted at ∆*T* ≥ 30 °C), the maximum packing fraction is met at shallow depths (< 500 m) during dike propagation (Fig. 6c, blue and red line). At that point, dike propagation is inhibited, with a rapid and sharp increase in viscosity of several orders of magnitude (Fig. 6d, blue and red line). According to our results, dike propagation will stop at ~500 m below the surface considering the maximum aspect ratio and closer to the surface (~50 m below the surface) assuming the minimum aspect ratio. Indeed, the dramatic increase in viscosity obtained assuming dendritic crystallisation (≥1 × 10^6 ^Pa s^−1^; Fig. 6d, blue and red line) results in a strong decrease in dike propagation velocity (Fig. 6e, blue and red line) that approaches 0 below the surface. Assuming an interface-controlled growth (*r*_p_ = 4; Fig. 6b, black line), instead, the increase in viscosity (<1 × 10^5 ^Pa s^−1^; Fig. 6d, black line) is not enough to prevent dike to reach the surface. Therefore, in this case magma can rise up to the surface and may be eruptible.

From the ascent velocity, we calculated the ascent time of the dike (Fig. 6f), estimating that the dike will reach the surface or stop after 4.5 h. This time of propagation may provide enough time to develop the maximum aspect ratio during dendritic growth (Fig. 3), and therefore the scenario where dike stops at ~500 m below the surface might be the most likely. Furthermore, considering that shear rate can further reduce the nucleation delay and accelerate the diffusion-limited growth process, the formation of large dendritic crystals can have a significant effect in the increase of viscosity preventing a magma in reaching the surface^8^.

**Fig. 7:**
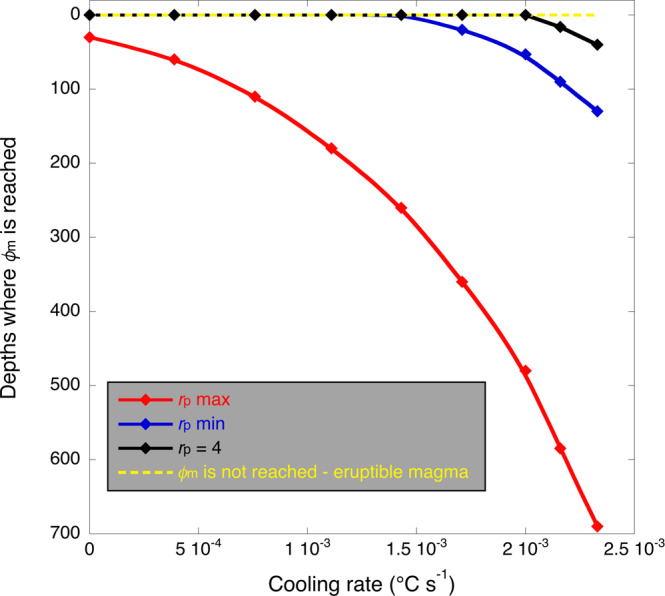
Depths where the maximum packing fraction (*ϕ*_m_) is achieved. Considering a constant aspect ratio (*r*_p_ = 4), typical of euhedral crystallization (black line), *ϕ*_m_ will not be achieved at depth and dike can propagate to the surface feeding an eruption. Only with cooling rates >2 × 10^−3^ °C s^−1^, the maximum packing fraction may be achieved close to the surface. Considering minimum aspect ratios (*r*_p_ min) achievable at ∆*T* ≥ 30 °C, typical of incipient dendritic crystallization (blue line), *ϕ*_m_ is achieved at depth only if cooling rates >1.5 × 10^−3^ °C s^−1^. Considering maximum aspect ratios (*r*_p_ max), typical of mature dendritic crystallization (red line), *ϕ*_m_ will be intersected at depth independently of the cooling rate. The yellow dashed line indicates the conditions where *ϕ*_m_ is not achieved and magma is eruptible. Data plotted in Fig. 7 are provided in the Supplementary Table 4.

The results of our model indicate that, when interface-controlled growth is promoted and euhedral crystals are formed (*r*_p_ = 4; black line in Fig. 7) at cooling rates ≤2 × 10^−3^ °C s^−1^, magma viscosity will be ≤10^4 ^Pa s^−1^ during magma ascent and the maximum packing fraction will not be reached at depth (Fig. 7). Only when cooling rates are >2 × 10^−3^ °C s^−1^ the maximum packing fraction for euhedral crystals can be achieved close to the surface (tens of metres below the surface) (black line in Fig. 7). If we consider incipient dendritic crystallization and minimum aspect ratios (blue line in Fig. 7) promoted by an initial strong disequilibrium dendritic crystal growth (i.e. the case of a rapid dike propagation), the maximum packing fraction is met only at cooling rates >1.5 × 10^−3^ °C s^−1^. Therefore, at cooling rates <1.5 × 10^−3^ °C s^−1^ if euhedral and incipient dendritic crystallization occur (with small aspect ratio dendritic crystals) during magma ascent, a dike can propagate up to the surface and feed eruptions (Fig. 7). This happened for example during the Mt Etna 2001 eruption^59^. Indeed, the textures of scoriae erupted from the dike-fed Mt Etna 2001 lava fountaining eruption, show euhedral phenocrysts and an incipient dendritic crystallization in microlites (Supplementary Fig. 5), in line with the results from our 4D experiments and numerical model predictions. If dendritic growth has enough time to reach the maximum aspect ratio (i.e. slow dike propagation, red line in Fig. 7), the maximum packing fraction will be met at depth considering all the cooling rates investigated (Fig. 7). In this case, our results show that the maximum packing fraction will be met deeper in the crust at higher cooling rates (Fig. 7), as higher ∆*T*s will be reached faster and at deeper depths. This may prevent eruptions as the magma can be locked within the crust, such as the Mt Etna dike studied previously by Mollo et al.^9^.

The integration of our 4D experiments on magma crystallization with results from dike-propagation modelling highlights the role of dendritic crystal growth on migration of basaltic magmas within the crust, since dendritic crystallization may affect magma viscosity and mobility, potentially freezing magma in a dike or reservoir within a few hours. Indeed, relatively modest cooling of the system may increase the undercooling enough to drive rapid dendritic crystallization, promote a transition from Newtonian to non-Newtonian rheological behaviour, and lock the magma within the shallow crust. Dendritic crystallization may also affect subsurface magma flow dynamics increasing its viscosity during ongoing dike eruptions, ultimately favouring eruption cessation. Finally, we should consider that modest heating of a magma stagnating in the crust, induced by multiple intrusions of fresh hot magma from depth, may reduce the degree of undercooling of the stored magma, switching the crystallization regime back from diffusion-limited growth to interface-controlled growth. This will unlock magma within the shallow crust, feasibly enabling an eruption to occur. Our experimental results indicate that the transition between the two crystallization regimes can happen within a temperature range of 10–30 °C. Crystallization under these conditions should be considered and investigated further, as it has strong implications for the locking and unlocking of basaltic magmas, affecting their mobility, eruptibility as well as feeding into volcanic risk assessment and mitigation models and management in active volcanic areas.

## Methods

### Starting material

The starting material used for our crystallization experiments is a trachybasalt from the lower vents of the 2001 Mt Etna eruption^21,37,59^. The anhydrous glassy starting material (Supplementary Table 1) was synthesised by melting a crushed rock sample in a Pt crucible. Melting was performed in a Nabertherm® MoSi_2_ box furnace at 1400 °C and at atmospheric pressure. The melt was left in the furnace for 4 h to allow it to fully degas and to dissolve any crystals present. The melt was then quenched in air to glass, and this procedure was repeated twice to enhance homogenisation. Cores were drilled from the glass and sealed in Au_80_Pd_20_ capsules of 3 mm diameter with 0.94 wt.% added water, then melted above the liquidus at 100 MPa and 1200 °C for 4 h using a TZM cold seal pressure vessel apparatus at the School of Earth Sciences, University of Bristol, UK. The pressure medium consisted of argon with hydrogen added to impose and maintain a sample ƒO_2_ close to NNO + 1. This melting period above the liquidus allowed us to hydrate the starting material homogenously. Before performing in situ crystallization experiments, the amount of water dissolved within the starting glass was quantified by Fourier Transform Infrared (FTIR) spectroscopy using a Nicolet i10 spectrometer, a MCT detector and an extended range KBr beamsplitter. Using the density trend and Etna basalt extinction coefficient of Lesne et al.^68^, the molecular H_2_O peak at 5200 cm^−1^ gives 0.20 wt.% and the OH peak at 4500 cm^−1^ gives 0.74 wt.% water, a total of 0.94 wt.% with the correct species proportions for the total water content. Finally, hydrous glassy cylinders 2.6 mm in diameter and 4 mm in length were used for in situ 4D X-ray computed microtomography crystallization experiments.

### In situ MPHT synchrotron X-ray microtomography experimental apparatus

In situ moderate pressure, high temperature (MPHT) experiments were performed at the X-ray microtomography beamline I12-JEEP^69^, Diamond Light Source, Harwell, UK. We used the high-temperature resistance Alice furnace^42^, which was combined with the P2R uniaxial mechanical rig^40,41^ (Supplementary Fig. 1) in order to pressurise the hydrous starting glass and to perform for the first time in situ 4D crystallization experiments under water-saturated conditions at crustal pressures. The Alice furnace^42^ allows us to control the temperature precisely, heating and cooling the sample from 0.05 °Cs^−1^ to 0.4 °Cs^−1^. Temperature was measured with an R-type thermocouple positioned close to the sample in the middle of the furnace hot spot. The furnace hot spot with homogeneous temperature measures a volume of ~4 × 4 × 4 mm^3^, and our samples were positioned within this area. The R-type thermocouple measures the sample temperature with an uncertainty of ±5 °C. The sample holder was an alumina crucible (Supplementary Fig. 1), which is suitable for the temperature range investigated and has a low X-ray attenuation coefficient. The crucible was mounted on the rotation stage of the P2R rig aligned with the customised piston (Supplementary Fig. 1). The P2R rig controlled the high-speed rotation at a fixed angle (3.125 deg s^−1^), which allowed the fast X-ray microtomography acquisition. To the aim of performing MPHT experiments, we developed a customised crucible-piston system that can encapsulate the hydrous magmatic samples at relatively high pressures and temperatures. The hydrous glass cylinder was placed in the customised alumina crucible and pressurised via the customised piston (Supplementary Fig. 1). The pressure was transferred to the sample from the piston in order to maintain the water dissolved in the melt and simulate magmatic pressures in a volcanic plumbing system. The sizes of the customised alumina crucible are 2.5 mm wall thickness, 3 mm internal diameter 8 mm external diameter and 10 mm length. A platinum (Pt) jacket around the piston increases the total external diameter of the piston to 3 mm. The platinum jacket allows the crucible to be sealed once the piston is inside, thereby pressurising the sample. At the lower extremity of the piston, which is in contact with the sample during the experiment, the Pt jacket wraps a pre-oxidised nickel (Ni) disk, which buffers the redox conditions of the system. The Pt jacket limits the diffusion of Ni through the sample. We used a Ni disk of 0.009 g, pre-oxidised in order to buffer the oxygen fugacity in air conditions.

### Experimental strategy

At the beginning of the experiment the sample was heated to 900 °C at ambient pressure with a continuous heating of 0.4 °Cs^−1^. This condition corresponds to 100 °C above the glass transition temperature (~800 °C) of our starting material, allowing us to pressurise the trachybasaltic glass cylinder with the piston by applying a load of ~300 N under uniaxial compression. The condition just above the glass transition makes it possible to press the sample down to the bottom of the crucible. At this stage the sample fills all the space inside the crucible and the piston maintains the load applied constantly for the entire duration of the experiment. The pressure imposed by the piston allows us to prevent H_2_O exsolution from the trachybasaltic melt during heating to the initial temperature above the liquidus (1210 and 1180 °C). Experiments were held for 10 min at the initial melting conditions above the liquidus. Since the pressure plays a major control on the H_2_O solubility in silicate melts, we calculated the H_2_O saturation in our trachybasaltic melt composition as a function of pressure using the water solubility model of Lesne et al.^68^. Our trachybasaltic melt with ~0.94 wt% of H_2_O dissolved is water-saturated at ~10 MPa. Therefore, this pressure represents the onset of H_2_O exsolution and bubble formation. Once the initial temperature above the liquidus was reached, a constant displacement of the piston was applied at a rate of 1 µm s^−1^ to reduce the initial load applied (~300 N). This reduction of the initial load, controlled by the P2R, generates a decompression of the system. H_2_O exsolution and bubble formation occurred close to ~10 MPa. The displacement of the piston was stopped at the appearance of the first bubbles. The load imposed by the piston was maintained constant during all the duration of the experiment. After 10 min at the initial melting conditions above the liquidus, the initial temperature of Exp-A (1210 °C) was decreased to 1150 °C for 90 min and then to 1130 °C for 90 min (Fig. 1 and Supplementary Table 2). The initial temperature of Exp-B (1180 °C) was decreased to 1160 °C for 90 min, then to 1140 °C for 60 min and, finally, to 1110 °C for 30 min (Fig. 1 and Supplementary Table 2).

### In situ synchrotron X-ray microtomography acquisition

The X-ray microtomography beamline I12-JEEP^69^ (Diamond Light Source, Harwell, UK) allows us to perform experiments in phase-contrast mode, setting the sample-to-detector distance at 2200 mm in order to work in the edge-detection regime^70^ (Supplementary Table 5). The tomographic projections were acquired using a monochromatic X-ray beam with energy of 53 keV. In each scan, 1440 tomographic projections were acquired by the detector with equiangular steps (3.125 deg s^−1^) over a full rotation angle of 180° (Supplementary Table 5). The exposure time for the acquisition of each projection was 0.04 s (Supplementary Table 5), thus the temporal resolution of each scan is 58 s. The isotropic pixel size was 3.2 μm. The detector is a high-resolution imaging PCO edge camera with optical module 3, corresponding to a field of view of 8.0 mm × 7.0 mm. Scan acquisition started at the initial conditions above the liquidus (melting period) and covered the entire duration of the experiment.

### Image reconstruction

Tomographic projections were reconstructed into 32-bit slices using Diamond I12 in-house python codes, using the Gridrec algorithm^71,72^ (http://confluence.diamond.ac.uk/display/I12Tech/Reconstruction+scripts+for+time+series+tomography)^73,74^. The pre-processing pipeline includes centre of rotation calculation^72^ zinger removal, blob removal^73^ and regularisation-based ring removal^74^.

The reconstructed slices were converted to 8-bit raw format and stacked using ImageJ software^75^ to obtain volumes in which the isotropic voxel has an edge size of 3.2 μm. Reconstructed volumes of experiments Exp-A and Exp-B were then cropped using Avizo® software version 2019.1 (Termo Fisher Scientific) in order to select the volume of interest (VOI) (Supplementary Table 5) for quantitative 3D image analysis. The VOI was chosen carefully in order to select the majority of the sample and to visualise and quantify the onset of crystallization and crystal growth during the experiments. Therefore, we discarded the crucible walls (around and at the bottom of the sample) and the Pt jacket of the piston at the top of the sample, which produced image artifacts (e.g. ring artifact).

Three-dimensional visualisation (volume rendering) of the reconstructed volumes was obtained using the commercial software VGStudio 3.0 (Volume Graphics), which allowed us to make 3D textural observations of the pyroxene crystal shapes (Figs. 2 and 3 and Supplementary Movie 1).

### Image processing and segmentation

Segmentation is the process that allows separation of objects from the background to obtain binary volumes containing only the feature of interest. Segmentation of pyroxene crystals from the glassy matrix was performed using Avizo® software (Supplementary Table 6). The whole image processing protocol, including segmentation and pre- and post-segmentation processing, lasted up to 10 h per frame. Before segmentation a 3D Non Local Mean filter in Avizo® software was applied to smooth the greyscale input images; this allows us to better distinguish and segment pyroxene crystals from their glassy matrix, reducing the noise and the potential artefacts whilst preserving edges and the shapes of the objects. Segmentation of pyroxene crystals from the glassy matrix was operated in the 3D domain with Avizo® software by using manual bi-level greyscale thresholding based on the greyscale histogram of the selected VOIs and visual inspection of the slices in different directions (axial, coronal and sagittal). Owing to the edge enhancement produced by the edge-detection regime used during the X-ray microtomography acquisition, phase-contrast artefacts were produced at the edge of the few bubbles present in the samples. Some bubble edges were segmented together with pyroxene crystals during the manual bi-level greyscale thresholding. Therefore, the Remove Small Spot operation in Avizo® software was used to remove the smaller bubble edges in the binary images. The larger bubble edges were manually removed with Avizo® software in order to complete segmentation of the pyroxene crystals.

For the rheological calculations, the entrapped melt within dendritic crystals is considered an additional solid part of the crystal, which contributes to further increase the crystal volume. To consider the entrapped melt, we applied the Cube Closing morphological operation to the final segmented dataset which allows us to perform a 3D closing of the empty space between dendritic crystals using a structuring element matching with a square in 2D and a cube in 3D (iterative computation was used for improved performance).

### Image analysis

The reconstructed segmented 3D images were analysed using Avizo® software version 2019.1. Clinopyroxene crystals volumes and sizes were measured in the 3D domain to obtain the textural and kinetic parameters reported in Figs. 3–5. The sizes (maximum, medium and minimum length) of the clinopyroxene crystals were measured along the three crystallographic axes, using the Measure tool of Avizo® software. This allowed us to quantify the growth rates and the crystal aspect ratios (*r*_p_) directly in 3D through time. The sizes of 5 representative single clinopyroxene crystals nucleated at different times were tracked every 5 min (particle tracking) in order to quantify their shape evolution and growth rates through time and they are reported in Figs. 3 and 4. The incremental growth rate (Y_Li_) was estimated considering the maximum axis lengths (*L*_max_) of each clinopyroxene crystal. The incremental growth rate was calculated using the following equation^45^:

6 $${{{{{{\rm{Y}}}}}}}_{{{{{{\rm{Li}}}}}}}=\frac{(0.5{L}_{{\max }2}-0.5{L}_{{\max }1})}{({{{{{{\rm{t}}}}}}}_{2}-{{{{{{\rm{t}}}}}}}_{1})}$$

where *L*_max2_ and *L*_max1_ are the maximum lengths measured at experimental times t_2_ and t_1_ respectively. The longest and shortest axes of each single clinopyroxene in Exp-A (~30 crystals) and of ~30 clinopyroxene crystals in Exp-B were measured to calculate the crystal aspect ratio (*r*_p_ = longest axis/shortest axis)^4,58^.

The volume of all clinopyroxene crystals present in the VOI of Exp-A and Exp-B was measured every 5 min in order to track the evolution of the crystal volume fraction (*ϕ*) through time. The crystal volume fraction (*ϕ*) of clinopyroxene was obtained using the following equation^37^:

7 $$\phi =\frac{{V}_{{{{{{{\mathrm{Cpx}}}}}}}}}{{V}_{{{{{{{\mathrm{BFV}}}}}}}}}$$

where *V*_Cpx_ is the volume of clinopyroxene crystals for each frame and *V*_BFV_ is the bubble-free VOI (BFV) which consists of only the volume of glass and crystals removing the bubbles within the VOI^37^.

### Electron microprobe analysis

The starting material (glasses) and the samples obtained during in situ crystallization experiments were analysed with a JEOL JXA-8530F field emission electron microprobe at the Photon Science Institute, University of Manchester, UK. The operating conditions were as follows: 15 kV accelerating voltage, 10 nA beam current, and beam diameter of 10 or 5 μm. Na and K were measured first to minimise loss by volatilisation. Calibration standards were albite for Na, periclase for Mg, corundum for Al, fayalite for Fe, tephroite for Mn, apatite for P, sanidine for K, wollastonite for Ca and Si and rutile for Ti. The composition of the starting material is reported in Supplementary Table 1. The composition of the clinopyroxene crystals formed during in situ experiments are rported in the Supplementary Fig. 2.

Back-scattered electron (BSE) images of the scoriae erupted from the Mt Etna 2001 lava fountaining eruption (Supplementary Fig. 5) were collected using a JEOL JSM-6390LA FE-SEM at the Department of Earth and Environmental Sciences, University of Manchester, UK. We used an acceleration voltage of 15 kV and beam current of 10 nA.

### SEM Electron backscatter diffraction measurements

SEM-EBSD measurements were carried out at the Bayerisches Geoinstitut of the University of Bayreuth on a Zeiss Gemini 1530 FEG SEM equipped with a Nordlys II EBSD detector and the Oxford Aztec Software Package for data analysis. The accelerating voltage and the beam current were set to 20 keV and ca. 2 nA respectively, yielding diffraction patterns that could be reliably indexed with respect to their orientation in automatic mode using 12 reflections from each pattern. Measuring times were 0.2 seconds per data point.

A thin section of the sample Exp-B was prepared by mechanical polishing it with a final grid size of 0.25 μm. The thin section was then coated with a thin layer (3 nm) of carbon to reduce charging. Three areas of the section with large clinopyroxene crystals were scanned with a step size of 0.5 μm in order to resolve the interior of the clinopyroxene with sufficient resolution. About 100 large clinopyroxene crystals could be analysed with respect to their interior microstructure as shown in the Supplementary Fig. 4.

## Supplementary information

Description of additional Supplementary File

Supplementary Information

Supplementary Movie 1

## Data Availability

The authors declare that the data supporting the findings of this study are available within the article and its supplementary information file. The data that support the findings of this study are available from the corresponding author upon request. The authors declare that the data supporting the findings of this study are available within the paper and its supplementary information file. Source data are provided with this paper.

## Source data

Source Data

## Code availability

The authors declare that the data generated in this study using the dike propagation model have been deposited in the Figshare database [10.6084/m9.figshare.19426343].

## References

[1] Caricchi L (2007). Non-Newtonian rheology of crystal-bearing magmas and implications for magma ascent dynamics. Earth Planet. Sci. Lett..

[2] Costa A, Caricchi L, Bagdassarov N (2009). A model for the rheology of particle-bearing suspensions and partially molten rocks. Geochem. Geophys. Geosyst..

[3] Giordano, D., Polacci, M., Papale, P. & Caricchi, L. Rheological control on the dynamics of explosive activity in the 2000 summit eruption of Mt. Etna. *Solid Earth* **1**, 61–69 (2010).

[4] Vona A, Romano C, Dingwell DB, Giordano D (2011). The rheology of crystal-bearing basaltic magmas from Stromboli and Etna. Geochim. Cosmochim. Acta.

[5] Cimarelli, C., Costa, A., Mueller, S. & Mader, H. M. Rheology of magmas with bimodal crystal size and shape distributions: Insights from analog experiments. *Geochem. Geophys. Geosyst*. **12**, Q07024 (2011).

[6] Mader HM, Llewellin EW, Mueller SP (2013). The rheology of two-phase magmas: a review and analysis. J. Volcanol. Geother. Res..

[7] Chevrel MO (2015). Viscosity measurements of crystallizing andesite from T ungurahua volcano (Ecuador). Geochem. Geophys. Geosyst..

[8] Kolzenburg S, Giordano D, Hess KU, Dingwell DB (2018). Shear rate‐dependent disequilibrium rheology and dynamics of basalt solidification. Geophys. Res. Lett..

[9] Mollo S (2011). Cooling history of a dike as revealed by mineral chemistry: a case study from Mt. Etna volcano. Chem. Geol..

[10] Hammer J, Jacob S, Welsch B, Hellebrand E, Sinton J (2016). Clinopyroxene in post shield Haleakala ankaramite: 1. Efficacy of thermobarometry. Contrib. Mineral. Petrol..

[11] Welsch B (2016). Clinopyroxene in postshield Haleakala ankaramite: 2. Texture, compositional zoning and supersaturation in the magma. Contrib. Mineral. Petrol..

[12] Cashman KV, Sparks RSJ, Blundy JD (2017). Vertically extensive and unstable magmatic systems: a unified view of igneous processes. Science.

[13] Albert H (2019). Magma interactions, crystal mush formation, timescales, and unrest during caldera collapse and lateral eruption at ocean island basaltic volcanoes (Piton de la Fournaise, La Réunion). Earth Planet. Sci. Lett..

[14] Albert H, Larrea P, Costa F, Widom E, Siebe C (2020). Crystals reveal magma convection and melt transport in dyke-fed eruptions. Sci. Rep..

[15] Rivalta E, Taisne B, Bunger AP, Katz RF (2015). A review of mechanical models of dike propagation: Schools of thought, results and future directions. Tectonophysics.

[16] Faure F, Trolliard G, Nicollet C, Montel JM (2003). A developmental model of olivine morphology as a function of the cooling rate and the degree of undercooling. Contrib. Mineral. Petrol..

[17] Del Gaudio P (2010). Cooling rate-induced differentiation in anhydrous and hydrous basalts at 500 MPa: implications for the storage and transport of magmas in dikes. Chem. Geol..

[18] Jerram DA, Davis GR, Mock A, Charrier A, Marsh BD (2010). Quantifying 3D crystal populations, packing and layering in shallow intrusions: a case study from the Basement Sill, Dry Valleys, Antarctica. Geosphere.

[19] Welsch B, Faure F, Famin V, Baronnet A, Bachèlery P (2013). Dendritic crystallization: a single process for all the textures of olivine in basalts?. J. Petrol..

[20] Shea T, Lynn KJ, Garcia MO (2015). Cracking the olivine zoning code: distinguishing between crystal growth and diffusion. Geology.

[21] Arzilli F (2019). Magma fragmentation in highly explosive basaltic eruptions induced by rapid crystallization. Nat. Geosci..

[22] Chatterjee N, Bhattacharji S, Fein C (2005). Depth of alkalic magma reservoirs below Kolekole cinder cone, Southwest rift zone, East Maui, Hawaii. J. Volcanol. Geotherm. Res..

[23] Neave DA, Maclennan J, Hartley ME, Edmonds M, Thordarson T (2014). Crystal storage and transfer in basaltic systems: the Skuggafjöll eruption, Iceland. J. Petrol..

[24] Gonnermann, H. & Taisne, B. *The Encyclopedia Of Volcanoes*. p. 215-224 (Academic Press, 2015).

[25] Kirkpatrick RJ (1981). Kinetics of crystallization of igneous rocks. Rev. Mineral. Geochem..

[26] Hammer JE (2008). Experimental studies of the kinetics and energetics of magma crystallization. Rev. Mineral. Geochem..

[27] Donaldson CH (1976). An experimental investigation of olivine morphology. Contrib. Mineral. Petrol..

[28] Hammer JE, Sharp TG, Wessel P (2010). Heterogeneous nucleation and epitaxial crystal growth of magmatic minerals. Geology.

[29] Lofgren, G. in *Physics of Magmatic Processes* (ed. Hargraves, R. B.) p. 487–551 (Princeton University, 1980).

[30] Pontesilli A (2019). Crystallization kinetics of clinopyroxene and titanomagnetite growing from a trachybasaltic melt: new insights from isothermal time-series experiments. Chem. Geol..

[31] Masotta M (2020). The role of undercooling during clinopyroxene growth in trachybasaltic magmas: Insights on magma decompression and cooling at Mt. Etna volcano. Geochim. Cosmochim. Acta.

[32] Moschini P (2021). Parameterization of clinopyroxene growth kinetics via crystal size distribution (CSD) analysis: Insights into the temporal scales of magma dynamics at Mt. Etna volcano. Lithos.

[33] Zhou JS (2021). Crystal growth of clinopyroxene in mafic alkaline magmas. Earth Planet. Sci. Lett..

[34] Neave DA, Putirka KD (2017). A new clinopyroxene-liquid barometer and implications for magma storage pressures under Icelandic rift zones. Am. Mineral..

[35] Ubide T, Kamber BS (2018). Volcanic crystals as time capsules of eruption history. Nat. Comm..

[36] Ubide T, Mollo S, Zhao JX, Nazzari M, Scarlato P (2019). Sector-zoned clinopyroxene as a recorder of magma history, eruption triggers, and ascent rates. Geochim. Cosmochim. Acta.

[37] Polacci M (2018). Crystallisation in basaltic magmas revealed via in situ 4D synchrotron X-ray microtomography. Sci. Rep..

[38] Le Gall N (2021). In situ quantification of crystallisation kinetics of plagioclase and clinopyroxene in basaltic magma: Implications for lava flow. Earth Planet. Sci. Lett..

[39] Lister JR, Kerr RC (1991). Fluid‐mechanical models of crack propagation and their application to magma transport in dykes. J. Geophys. Res. Solid Earth.

[40] Puncreobutr C, Lee PD, Hamilton RW, Phillion AB (2012). Quantitative 3D characterization of solidification structure and defect evolution in Al alloys. JOM.

[41] Puncreobutr C, Lee PD, Hamilton RW, Cai B, Connolley T (2013). Synchrotron tomographic characterization of damage evolution during aluminum alloy solidification. Metall. Mater. Trans. A.

[42] Azeem MA (2017). Revealing dendritic pattern formation in Ni, Fe and Co alloys using synchrotron tomography. Acta Mater..

[43] Gualda GA, Ghiorso MS, Lemons RV, Carley TL (2012). Rhyolite-MELTS: a modified calibration of MELTS optimized for silica-rich, fluid-bearing magmatic systems. J. Petrol..

[44] Kouchi A, Sugawara Y, Kashima K, Sunagawa I (1983). Laboratory growth of sector zoned clinopyroxenes in the system CaMgSi_2_O_6_-CaTiAl_2_O_6_. Contrib. Mineral. Petrol..

[45] Arzilli F, Carroll MR (2013). Crystallization kinetics of alkali feldspars in cooling and decompression-induced crystallization experiments in trachytic melt. Contrib. Mineral. Petrol..

[46] Vernon, R. H. *A Practical Guide To Rock Microstructure* (Cambridge university press, 2018).

[47] Faure F, Schiano P, Trolliard G, Nicollet C, Soulestin B (2007). Textural evolution of polyhedral olivine experiencing rapid cooling rates. Contrib. Mineral. Petrol..

[48] Clark AH, Pearce TH, Roeder PL, Wolfson I (1986). Oscillatory zoning and other microstructures in magmatic olivine and augite: Nomarski interference contrast observations on etched polished surface. Am. Mineral..

[49] Mollo S, Hammer JE (2017). Dynamic crystallization in magmas. EMU Notes Miner..

[50] Salas P, Ruprecht P, Hernández L (2021). Out-of-sequence skeletal growth causing oscillatory zoning in arc olivines. Nat. Commun..

[51] Sato H (2005). Viscosity measurement of subliquidus magmas: 1707 basalt of Fuji volcano. J. Mineral. Petrol. Sci..

[52] Ishibashi H (2009). Non-Newtonian behavior of plagioclase-bearing basaltic magma: subliquidus viscosity measurement of the 1707 basalt of Fuji volcano, Japan. J. Volcanol. Geotherm. Res..

[53] Ishibashi H, Sato H (2007). Viscosity measurements of subliquidus magmas: alkali olivine basalt from the Higashi-Matsuura district, Southwest Japan. J. Volcanol. Geotherm. Res..

[54] Soldati A, Farrell JA, Sant C, Wysocki R, Karson JA (2020). The effect of bubbles on the rheology of basaltic lava flows: Insights from large-scale two-phase experiments. Earth Planet. Sci. Lett..

[55] Kolzenburg S, Giordano D, Di Muro A, Dingwell DB (2019). Equilibrium viscosity and disequilibrium rheology of a high magnesium basalt from piton De La Fournaise volcano, La Reunion, Indian Ocean, France. Ann. Geophys..

[56] Kolzenburg S, Hess KU, Berlo K, Dingwell DB (2020). Disequilibrium rheology and crystallization kinetics of basalts and implications for the Phlegrean volcanic District. Front. Earth Sci..

[57] Krieger IM, Dougherty TJ (1959). A mechanism for non-Newtonian flow in suspensions of rigid spheres. Trans. Soc. Rheol..

[58] Mueller S, Llewellin EW, Mader HM (2010). The rheology of suspensions of solid particles. Proc. Math. Phys. Eng. Sci..

[59] Corsaro RA, Miraglia L, Pompilio M (2007). Petrologic evidence of a complex plumbing system feeding the July-August 2001 eruption of Mt. Etna, Sicily, Italy. Bull. Volcanol..

[60] Bonaccorso A, Currenti G, Del Negro C, Boschi E (2010). Dike deflection modelling for inferring magma pressure and withdrawal, with application to Etna 2001 case. Earth Planet. Sci. Lett..

[61] Holness MB (2014). The effect of crystallization time on plagioclase grain shape. Contrib. Mineral. Petrol..

[62] La Spina G, Burton M, Vitturi MDM (2015). Temperature evolution during magma ascent in basaltic effusive eruptions: A numerical application to Stromboli volcano. Earth Planet. Sci. Lett..

[63] La Spina G, Burton M, Vitturi MDM, Arzilli F (2016). Role of syn-eruptive plagioclase disequilibrium crystallization in basaltic magma ascent dynamics. Nat. Commun..

[64] La Spina G (2021). Explosivity of basaltic lava fountains is controlled by magma rheology, ascent rate and outgassing. Earth Planet. Sci. Lett..

[65] Delaney PT, Pollard DD (1982). Solidification of basaltic magma during flow in a dike. Am. J. Sci..

[66] Ujike O (1982). Microprobe mineralogy of plagioclase, clinopyroxene and amphibole as records of cooling rate in the Shirotori—Hiketa dike swarm, northeastern Shikoku, Japan. Lithos.

[67] Blundy J, Cashman K, Humphreys M (2006). Magma heating by decompression-driven crystallization beneath andesite volcanoes. Nature.

[68] Lesne P, Scaillet B, Pichavant M, Iacono-Marziano G, Beny JM (2011). The H_2_O solubility of alkali basaltic melts: an experimental study. Contrib. Mineral. Petrol..

[69] Drakopoulos M (2015). I12: the joint engineering, environment and processing (JEEP) beamline at diamond light source. J. Synchrotron Radiat..

[70] Cloetens P, Barrett R, Baruchel J, Guigay JP, Schlenker M (1996). Phase objects in syn- chrotron radiation hard X-ray imaging. J. Phys. D Appl. Phys..

[71] O’sullivan JD (1985). A fast sinc function gridding algorithm for Fourier inversion in computer tomography. IEEE Trans. Med. Imaging.

[72] Gürsoy D, De Carlo F, Xiao X, Jacobsen C (2014). TomoPy: a framework for the analysis of synchrotron tomographic data. J. Synchrotron Radiat..

[73] Vo NT, Drakopoulos M, Atwood RC, Reinhard C (2014). Reliable method for calculating the center of rotation in parallel-beam tomography. Opt. Express.

[74] Vo NT, Atwood RC, Drakopoulos M (2018). Superior techniques for eliminating ring artifacts in X-ray micro-tomography. Opt. Express.

[75] Abràmoff MD, Magalhães PJ, Ram SJ (2004). Image processing with ImageJ. Biophoton. Int..

