# Supplementary material for: Dendritic crystallization in hydrous basaltic magmas controls magma mobility within the Earth’s crust
Source: Nat Commun. 2022 Jun 10;13:3354. doi: 10.1038/s41467-022-30890-8 (PMC9187734; doi:10.1038/s41467-022-30890-8)
Supplement: Supplementary file 1 — Description of additional Supplementary File [file 41467_2022_30890_MOESM1_ESM.pdf]

Descriptions of Additional Supplementary Data Files

Supplementary Movie 1 : Crystallization and crystal shape evolution of a clinopyroxene crystal through time during the experiment Exp-A.
