# Supplementary material for: Dendritic crystallization in hydrous basaltic magmas controls magma mobility within the Earth’s crust
Source: Nat Commun. 2022 Jun 10;13:3354. doi: 10.1038/s41467-022-30890-8 (PMC9187734; doi:10.1038/s41467-022-30890-8)
Supplement: Supplementary file 2 — Supplementary Information [file 41467_2022_30890_MOESM2_ESM.pdf]

Supplementary Information

Dendritic crystallization in hydrous basaltic magmas controls magma mobility within the Earth's crust

Fabio Arzilli[1,2*], Margherita Polacci[2], Giuseppe La Spina[3], Nolwenn Le Gall[4,5], Edward W. Llewellin[6], Richard A. Brooker[7], Rafael Torres-Orozco[8,9], Danilo Di Genova[10], David A. Neave[2], Margaret E. Hartley[2], Heidy M. Mader[7], Daniele Giordano[11], Robert Atwood[12], Peter D. Lee[4,5], Florian Heidelbach[10] and Mike R. Burton[2]

[1]School of Science and Technology, Geology Division, University of Camerino, Camerino, Italy

[2]Department of Earth and Environmental Sciences, University of Manchester, Manchester M13 9PL, UK

[3]Istituto Nazionale di Geofisica e Vulcanologia-Osservatorio Etneo, Sezione di Catania, Catania, Italy

[4]Department of Mechanical Engineering, University College London, London, UK

[5]Research Complex at Harwell, Rutherford Appleton Laboratory, Harwell, Oxfordshire, 11 OX11 0FA, UK

[6]Department of Earth Sciences, Durham University, Durham DH1 3LE, UK

[7]School of Earth Sciences, University of Bristol, Bristol BS8 1RJ, UK

[8]Centro de Ciencias de la Tierra, Universidad Veracruzana, Xalapa 91090, Mexico

[9]Centre of Geosciences, National Autonomous University of Mexico, Queretaro 76230, Mexico

[10]Bayerisches Geoinstitut, University of Bayreuth, 95440 Bayreuth, Germany

[11]Department of Earth Science, University of Torino, 10125 Torino, Italy

[12]Diamond Light Source, Harwell Science and Innovation Campus, Didcot OX11 0DE, UK

[*]Corresponding author: Dr. Fabio Arzilli[1,2]

E-mail address: fabio.arzilli@unicam.it; Phone: +393298429732; +447904104670

Supplementary Figure 1. Experimental apparatus. The P2R uniaxial mechanical rig was combined with a high-temperature environmental cell (Alice furnace) to perform moderate pressure, high temperature crystallization experiments. These two apparatuses combined with fast synchrotron X-ray microtomography (at beamline I12-JEEP, Diamond Light Source, Harwell, UK) allowed us to capture the evolution of crystallization in real time.

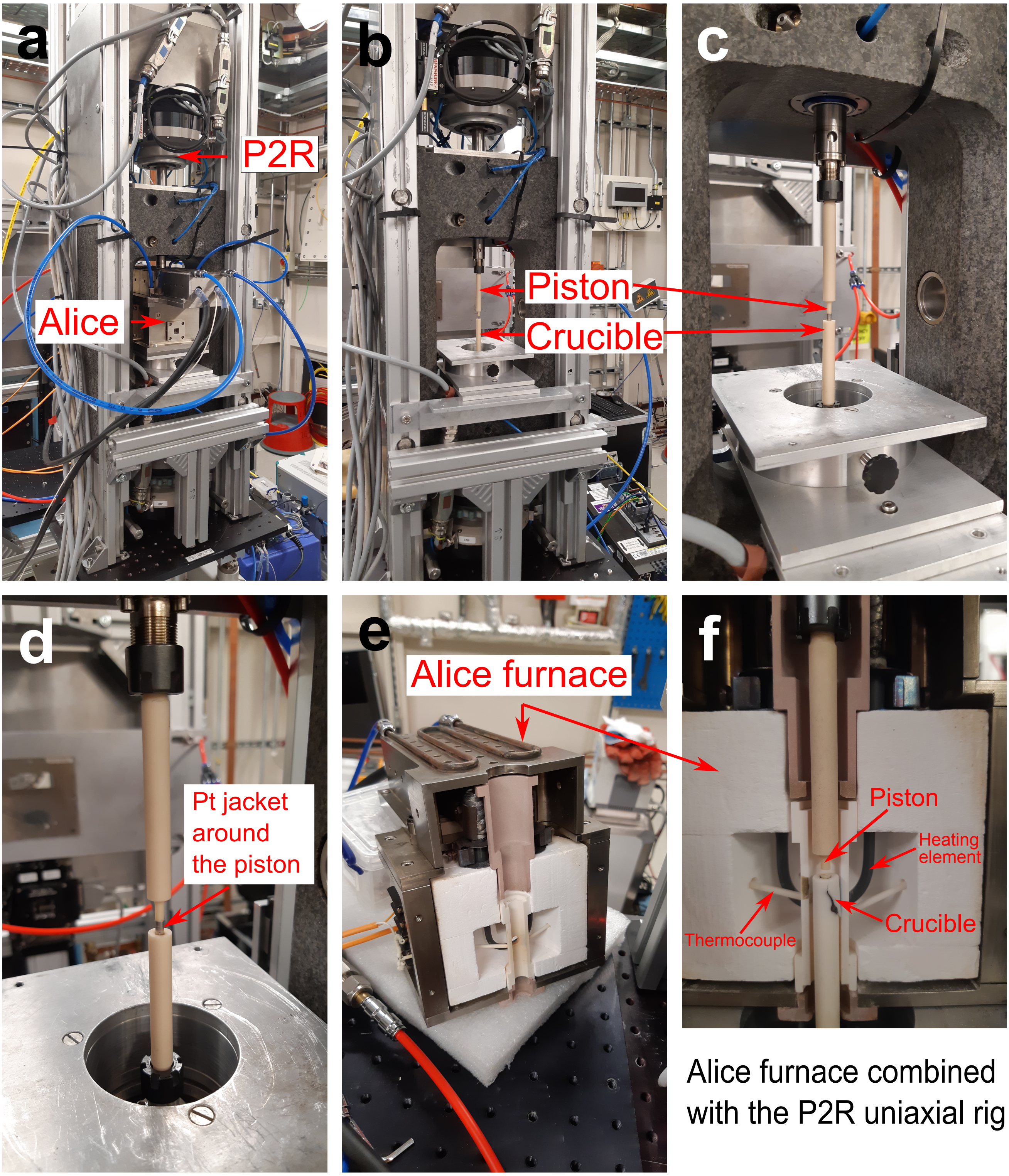

Supplemetary Figure 2. Chemical compositions of clinopyroxene crystals. Compositions of clinopyroxene crystals formed during the experiment Exp-B. Clinopyroxene crystals ot the experiment Exp-B are compared with those erupted from the lava fountaining of the Etna 2001 eruption. Blue square=Exp-B; red diamond=Etna 2001 eruption. En: enstatite; Fs: ferrosilite; Di: diopside; Hd: hedenbergite; Wo: Wollastonite.

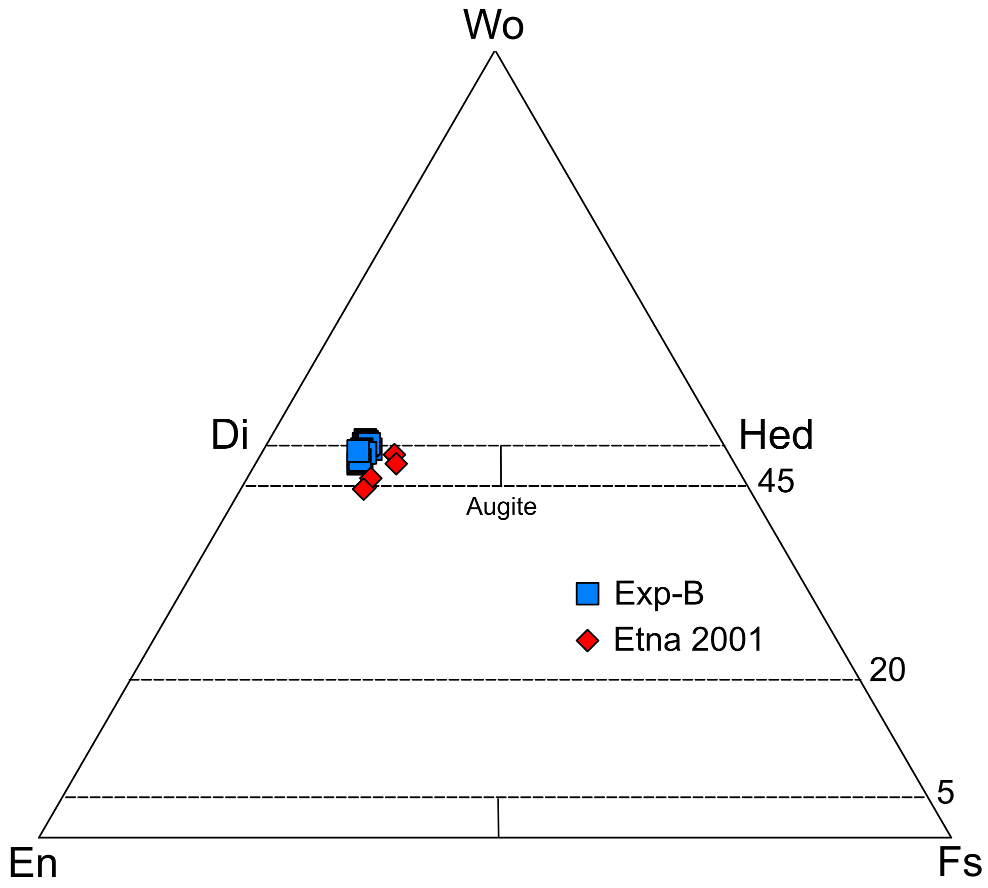

Supplementary Figure 3. Crystallization through time during experiments Exp-A and Exp-B. Reconstructed axial slices show crystallization of a hydrous trachybasaltic melt through time during single step cooling experiments at 10 MPa. **a**, **b**, **c**, Reconstructed axial slices show clinopyroxene crystallization through time during the experiment Exp-A at 1150 °C and ΔT=10 °C after 31 minutes (**a**), 51 minutes (**b**) and 71 minutes (**c**) from the beginning of the experiment. The temperature is decreased from 1150 °C to 1130 °C at the minute 90th. **d**, **e**, **f**, Reconstructed axial slices show clinopyroxene crystallization through time during the experiment Exp-A at 1130 °C and ΔT=30 °C after 91 minutes (**d**), 111 minutes (**e**) and 121 minutes (**f**) from the beginning of the experiment. **g**, **h**, **i**, Reconstructed axial slices show clinopyroxene crystallization through time during the experiment Exp-B at 1140 °C and ΔT=20 °C after 31 minutes (**g**), 41 minutes (**h**) and 51 minutes (**i**) from the beginning of the experiment. The temperature is decreased from 1150 °C to 1130 °C at the minute 60th **j**, **k**, **l**, Reconstructed axial slices show clinopyroxene crystallization through time during the experiment Exp-B at 1110 °C and ΔT=50 °C after 66 minutes (**j**), 76 minutes (**k**) and 86 minutes (**l**) from the beginning of the experiment. The red and yellow circles highlight the transition from euhedral (**d** and **j**) to dendritic (**e**,**f**,**k**,**l**) shapes of two clinopyroxene crystals. Cpx = clinopyroxene; bl: bubbles.

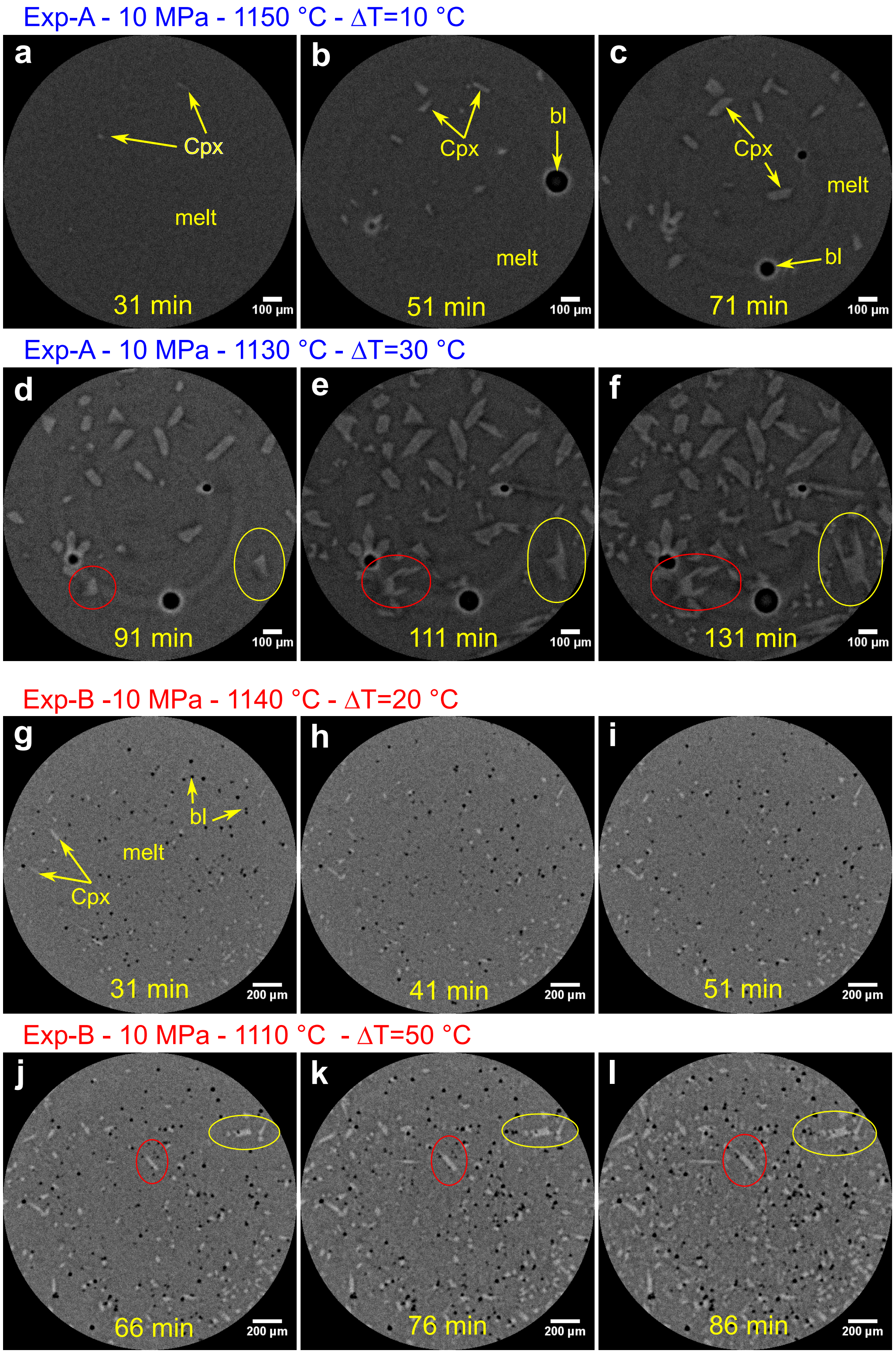

Supplementary Figure 4. Electron backscatter diffraction (EBSD) results. EBSD data indicate that the vast majority of the clinopyroxene crystals has a low internal misorientation (between 0 and 2°); only some crystals show a continuous lattice bending up to 3°. Rarely a larger stepwise misorientation (<6°) between host crystal and branch in the form of a subgrain boundary is observed. The EBSD results suggest that the branches growing from the corners of originally euhedral crystals are in crystallographic continuity with the central part of the crystal, therefore, the branches are not new crystals heterogeneously nucleated from the euhedral crystal, but are an elongate extension of the original grain.

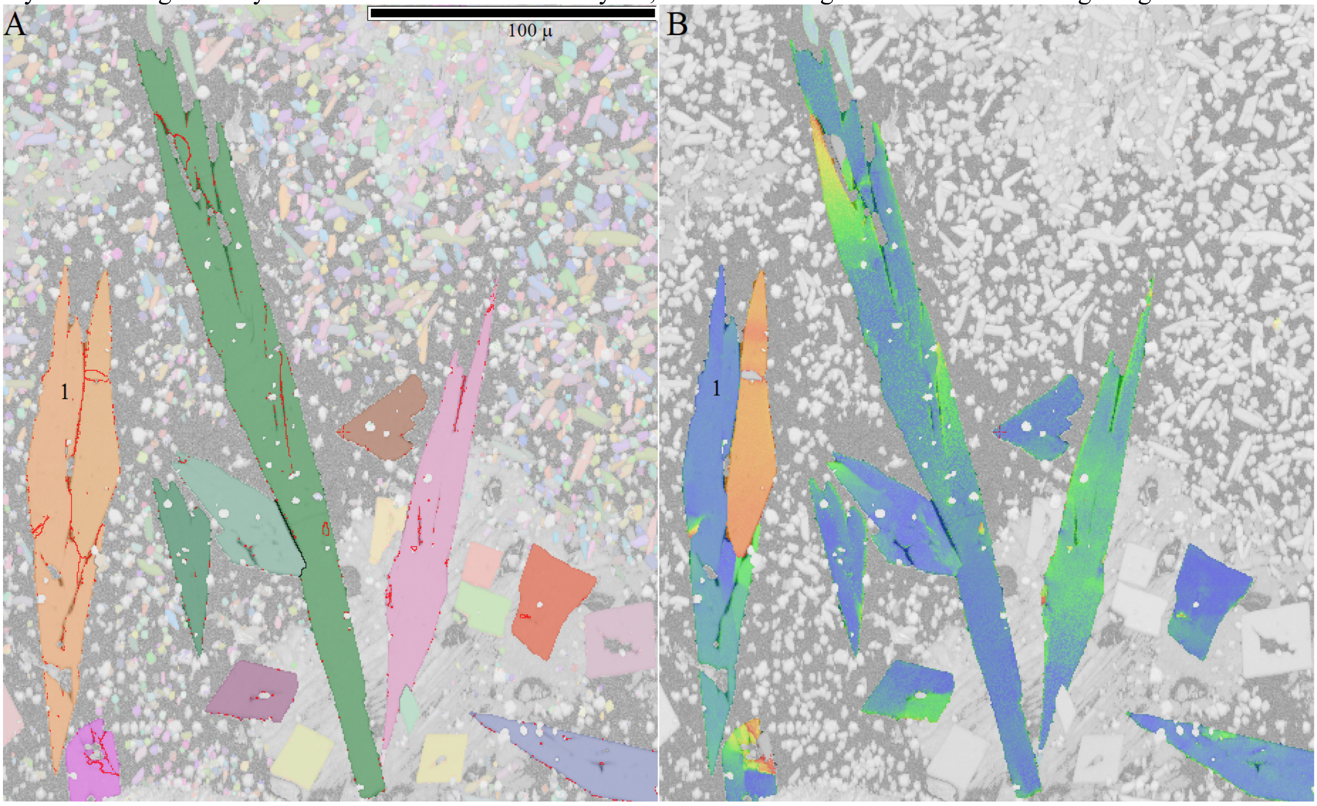

Supplemetary Figure 5. Textures of scoriae erupted from the Mt Etna 2001 lava fountaining eruption, fed by a dike intrusion. **a**, **b**, **c**, Back scattered images show euhedral plagioclase (Pl) and clinopyroxene (Cpx) phenocrysts. Yellow arrows indicate an incipient dendritic crystallization of plagioclase microlites.

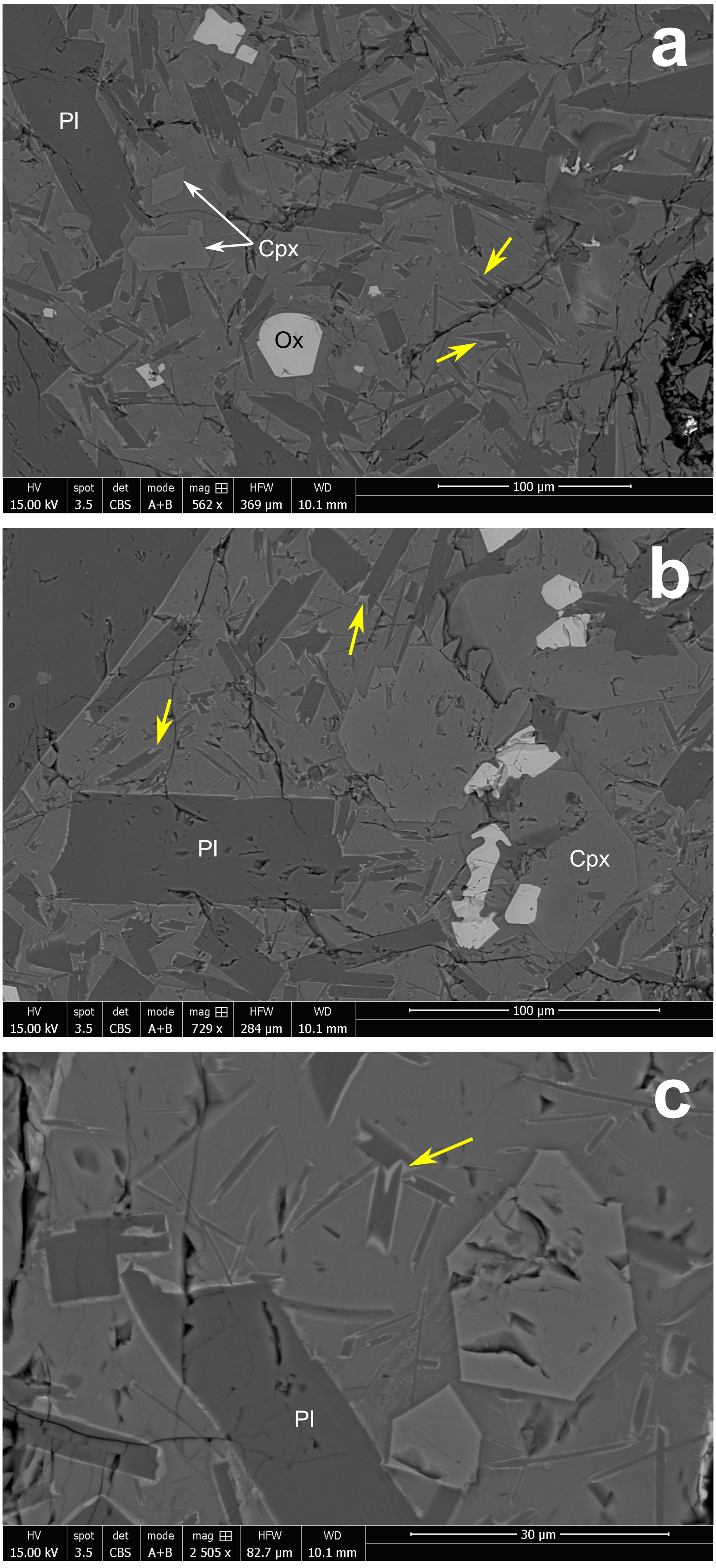

Supplementary Table 1. Chemical composition of the starting glass used for the experiments Exp-A and Exp-B.

Oxide (wt.%)	Starting bulk	st. dev.
SiO_2	48.41	0.16
TiO_2	1.79	0.07
Al_2O_3	16.22	0.50
FeO*	10.66	0.30
MnO	0.20	0.03
MgO	6.20	0.32
CaO	10.66	0.19
Na_2O	3.41	0.20
K_2O	1.90	0.07
P_2O_5	0.54	0.04
Total	99.85	

note: FeO* = total iron as FeO. The starting bulk composition is trachybasalt from the lower vents of the 2001 Etna eruption.

Supplementary Table 2. Experimental conditions for *in situ* experiments performed at beamline I12-JEEP (Diamond Light Source, Harwell, UK) combining the P2R uniaxial mechanical rig, Alice furnace and fast synchrotron X-ray microtomography.

Sample	P (Mpa)	T_i (°C)	T_1 (°C)	T_2 (°C)	T_3 (°C)	t_i (min)	t_1 (min)	t_2 (min)	t_3 (min)
Exp A	10	1210	1150	1130	-	10	90	60	-
Exp B	10	1180	1160	1140	1110	10	90	60	30

note: P = pressure; T_i = initial temperature above the liquidus; T_1 = temperature investigated after the first single step cooling from the initial temperature; T_2 = temperature investigated after the second single step cooling from T_1; T_3 = temperature investigated after the second single step cooling from T_2; t_i = dwell time at initial conditions; t_1 = dwell time at T_1; t_2 = dwell time at T_2; t_3 = dwell time at T_3.

Supplementary Table 3. 4D textural data.

Experiment	T (°C)	ΔT (°C)	time (min)	ϕ	r_p	ϕ_m	ϕ/ϕ_m	n
**Exp-A**								
Euhedral crystals fromed by interface-controlled growth								
	1150	10	26	1.19E-05	3.89	0.46	2.59E-05	1.00
	1150	10	31	1.55E-04	3.36	0.48	3.24E-04	1.00
	1150	10	36	3.67E-04	3.45	0.48	7.71E-04	1.00
	1150	10	41	6.20E-04	3.62	0.47	1.32E-03	1.00
	1150	10	46	9.47E-04	3.47	0.48	1.99E-03	1.00
	1150	10	51	1.29E-03	3.32	0.48	2.69E-03	1.00
	1150	10	56	2.04E-03	3.40	0.48	4.27E-03	1.00
	1150	10	61	3.46E-03	3.44	0.48	7.27E-03	1.00
	1150	10	66	4.33E-03	3.49	0.47	9.13E-03	1.00
	1150	10	71	0.01	3.24	0.48	0.01	1.00
	1150	10	76	0.01	3.22	0.48	0.01	1.00
	1150	10	81	0.01	3.24	0.48	0.01	1.00
	1150	10	86	0.01	3.19	0.48	0.02	1.00
Dendritic crystals fromed by diffusion-controlled growth								
	1130	30	91	0.01	3.11	0.49	0.02	1.00
	1130	30	96	0.01	4.40	0.45	0.02	1.00
	1130	30	101	0.01	5.81	0.41	0.03	1.00
	1130	30	106	0.02	6.90	0.39	0.06	1.00
	1130	30	111	0.03	7.69	0.37	0.08	1.00
	1130	30	116	0.04	9.62	0.34	0.12	1.00
	1130	30	121	0.06	11.00	0.32	0.20	1.00
	1130	30	126	0.08	12.03	0.31	0.25	0.99
	1130	30	131	0.09	12.31	0.30	0.31	0.98
	1130	30	136	0.10	12.38	0.30	0.32	0.97
	1130	30	141	0.10	12.35	0.30	0.34	0.97
	1130	30	146	0.11	12.63	0.30	0.37	0.95
Dendritic crystals + entraped melt within the dendrites								
	1130	30	91	0.01	3.11	0.49	0.02	1.00
	1130	30	96	0.01	4.40	0.45	0.02	1.00
	1130	30	101	0.01	5.81	0.41	0.03	1.00
	1130	30	106	0.03	6.62	0.39	0.07	1.00
	1130	30	111	0.04	7.21	0.38	0.11	1.00
	1130	30	116	0.06	9.52	0.34	0.17	1.00
	1130	30	121	0.09	10.46	0.33	0.27	0.99
	1130	30	126	0.11	12.04	0.31	0.35	0.96
	1130	30	131	0.13	12.30	0.30	0.43	0.92
	1130	30	136	0.13	12.44	0.30	0.45	0.90
	1130	30	141	0.15	12.40	0.30	0.49	0.86
	1130	30	146	0.16	12.67	0.30	0.53	0.80
**Exp-B**								
Euhedral crystals fromed by interface-controlled growth								
	1140	20	21	1.55E-03	2.00	0.53	0.00	1.00
	1140	20	26	4.12E-03	2.41	0.51	0.01	1.00
	1140	20	31	0.01	3.64	0.47	0.02	1.00
	1140	20	36	0.01	4.21	0.45	0.03	1.00
	1140	20	41	0.01	4.69	0.44	0.03	1.00
	1140	20	46	0.02	5.48	0.42	0.05	1.00
	1140	20	51	0.02	6.14	0.40	0.05	1.00
	1140	20	56	0.02	5.97	0.41	0.06	1.00
Dendritic crystals fromed by diffusion-controlled growth								
	1110	50	61	0.03	5.50	0.42	0.07	1.00
	1110	50	66	0.04	5.14	0.43	0.08	1.00
	1110	50	71	0.04	5.74	0.41	0.10	1.00
	1110	50	76	0.05	7.07	0.38	0.13	1.00
	1110	50	81	0.05	9.17	0.35	0.16	1.00
	1110	50	86	0.06	10.52	0.33	0.18	1.00
Dendritic crystals + entraped melt within the dendrites								
	1110	50	91	0.03	5.50	0.42	0.07	1.00
	1110	50	96	0.04	5.14	0.43	0.08	1.00
	1110	50	101	0.04	5.74	0.41	0.10	1.00
	1110	50	106	0.06	7.07	0.38	0.15	1.00
	1110	50	111	0.07	9.17	0.35	0.21	1.00
	1110	50	116	0.09	10.52	0.33	0.27	0.99

note: T = experimental temperature; ΔT = undercooling; time = dwell time at constant temperature; ϕ = crystal fraction; r_p = crystal aspect ratio; ϕ_m = maximum packing fraction; n = flow index. Exp-A: the onset of clinopyroxene crystallization occurred after 21 minutes at 1150 °C (ΔT = 10 °C) during experiment Exp-A, but the crystals were segmentable after 26 minutes. Exp-B: the onset of clinopyroxene crystallization occurred after 5 minutes at 1140 °C (ΔT = 20 °C) during experiment Exp-B, but the crystals were segmentable after 21 minutes.

Supplementary Table 4. Data of the dike propagation model obtained investigating depths where the maximum packing fraction (ϕ_m) is achieved.

cooling rate (°C/s)	Depths where ϕ_m is reached		
	r_p max	r_p min	r_p = 4
0.00E+00	30	0	0
3.90E-04	60	0	0
7.60E-04	110	0	0
1.11E-03	180	0	0
1.43E-03	260	0	0
1.71E-03	360	20	0
2.00E-03	480	53	0
2.16E-03	585	90	16
2.33E-03	690	130	40

note: ϕ_m = maximum packing fraction; r_p = aspect ratio.

Supplementary Table 5. X-ray microtomography acquisition conditions.

Sample	Energy	Distance sample to detector	Projection	Exposure time	Voxel size	Original imaged volume
	keV	mm	nember	s	µm (µm^3)	pixels (mm^3)
Exp A	53	2200	1440	0.04	3.2x3.2x3.2 (32.8)	1185x1185x800 (36.8)
Exp B	53	2200	1440	0.04	3.2x3.2x3.2 (32.8)	1185x1185x800 (36.8)

note: Original imaged volume: the volume includes the sample, the piston and the crucible.

Supplementary Table 6. Image processing and segmentation.

Sample	Analyzed VOI	Software	Pre-segmetation processing	Segmentation	Post-segmetation processing
	mm^3		Edge-preserving filter	Threshold	Remove outliers operation
Exp A	0.7	Avizo	3D Non Local Mean	manual bi-level greyscale thresholding	Remove Small Spot
Exp B	1.1	Avizo	3D Non Local Mean	manual bi-level greyscale thresholding	Remove Small Spot

note: VOI: the volume of interest was selected for quantitative 3D image analysis. The volume of interest consists of the majority part of the sample, which allows us to visualize and quantify crystallization through time with quantitative 3D image analysis. We discarded the crucible walls (around and at the bottom of the sample) and the piston (with its Pt jacket) at the top of the sample, which produced image artifacts. Avizo: Avizo® software version 2019.1 (Thermo Fisher Scientific).
